# Photoinduced ultrafast multielectron transfer and long-lived charge-accumulated state in a fullerene-indacenodithiophene dumbbell triad

**DOI:** 10.1073/pnas.2414671121

**Published:** 2024-12-05

**Authors:** Chong Wang, Bo Wu, Yang Li, Ying Jiang, Tianyang Dong, Shen Zhou, Chunru Wang, Chunli Bai

**Affiliations:** ^a^Beijing National Laboratory for Molecular Sciences, Key Laboratory of Molecular Nanostructure and Nanotechnology, Institute of Chemistry, Chinese Academy of Sciences, Beijing 100190, China; ^b^University of Chinese Academy of Sciences, Beijing 100049, China; ^c^School of Science, Beijing University of Posts and Telecommunications, Beijing 100876, China; ^d^College of Science, Hunan Key Laboratory of Mechanism and Technology of Quantum Information, National University of Defense Technology, Changsha 410003, China

**Keywords:** fullerene, ultrafast multielectron transfer, long-lived charge-accumulated state, photocatalysis

## Abstract

Photoinduced ultrafast multielectron transfer (m-ET) and long-lived charge-accumulated states in single molecules hold promise for light-energy conversion and utilization. However, compared to single-electron transfer (s-ET), m-ET tends to be thermodynamically and kinetically unfavorable. Here, we construct a dumbbell-shaped fullerene-indacenodithiophene triad, **IT2**, modified with two C_60_ units in the donor indacenodithiophene. Exciting the C_60_ units, ultrafast m-ET occurs with a time constant of 0.5 ps, accumulating two holes with a lifetime of 10 μs. Benefitting from a larger driving force, lower reorganization energy, and smaller structural changes, the rate of m-ET is 23 times faster than that of s-ET, and the lifetime of the m-ET product is 1.4 × 10^5^ times longer than that of the s-ET products. These attributes endow **IT2** with superior photocatalytic performance in multielectron oxidation reactions. This is an instance of achieving faster m-ET and a longer m-ET product lifetime than s-ET in a single molecule. This finding provides unique insights for the construction and application of intramolecular m-ET and charge accumulation systems in photocatalysis and molecular devices.

## Significance

Multielectron transfer (m-ET) photoelectronic systems have garnered significant interest due to their enhanced suitability for photoreactions, though constructing such systems poses considerable challenges. In this work, a dumbbell-shaped fullerene-indacenodithiophene triad has been constructed, which achieves ultrafast m-ET with a time constant of 0.5 ps, 23 times faster than single-electron transfer (s-ET), and obtains long-lived m-ET product with a 10 μs lifetime, 1.4 × 10^5^ times longer than the s-ET products. This development promises significant advancements in the study and application of m-ET and charge accumulation systems in light-energy conversion, photocatalysis, photoelectronic devices, and other fields.

Multielectron-involved chemical reactions are prevalent in nature, such as water oxidation and CO_2_ reduction in natural photosynthetic centers (1–6). Inspired by this, achieving multielectron transfer (m-ET) by harnessing clean and renewable solar energy is of pivotal importance for charge accumulation, photocatalysis, and other fields (2, 7). Herein, ultrafast m-ET and long-lived charge-accumulated states (m-ET products) can provide more opportunities for the separated charges to participate in subsequent reactions, promoting better light-energy utilization and conversion (8). Significant progress has been made in these fields using donor–acceptor (D-A) structural organic photoelectronic materials (9–12). However, current photoelectronic materials mainly involve single-electron transfer (s-ET) after excited (13, 14), posing challenges for achieving multielectron photochemical transformations (15, 16). Therefore, the construction and application of m-ET materials, along with mechanism studies, are still worth exploring (2, 3, 14). This necessitates photoelectronic molecules that can be excited by multiple photons and transfer multiple electrons to achieve charge accumulation. Previous studies have reported the use of electron/hole sacrificial agents to complete multielectron redox reactions (17, 18). However, for sustainable solar energy conversion and utilization, achieving photoinduced m-ET in a single molecule without sacrificial reagents is more promising (7, 16). This approach can not only improve photocatalytic conversion efficiency but also has the potential to construct light-operated single-molecule devices for charge accumulation.

Constructing m-ET systems is a rigorous challenge, due to competition with charge recombination (CR) (3). Compounding the issue, the rate of m-ET is typically slower than that of s-ET, impeding further electron transfer since the donor or acceptor units of the s-ET products have already donated or accepted one electron (3). This creates thermodynamic and kinetic barriers to m-ET. To alleviate this, D-A-D or A-D-A structures can be employed, where multiple electrons can be transferred from different donors or acceptors after multiple chromophores are excited. Many successful examples employing this strategy have been reported (1, 16, 19, 20). However, the issues of slow m-ET rates and short product lifetimes still persist. To achieve more favorable m-ET kinetics, both energy and structural factors must be considered (21). Only when these factors are optimized is it possible to achieve ultrafast m-ET and long-lived m-ET products.

**Fig. 1.**
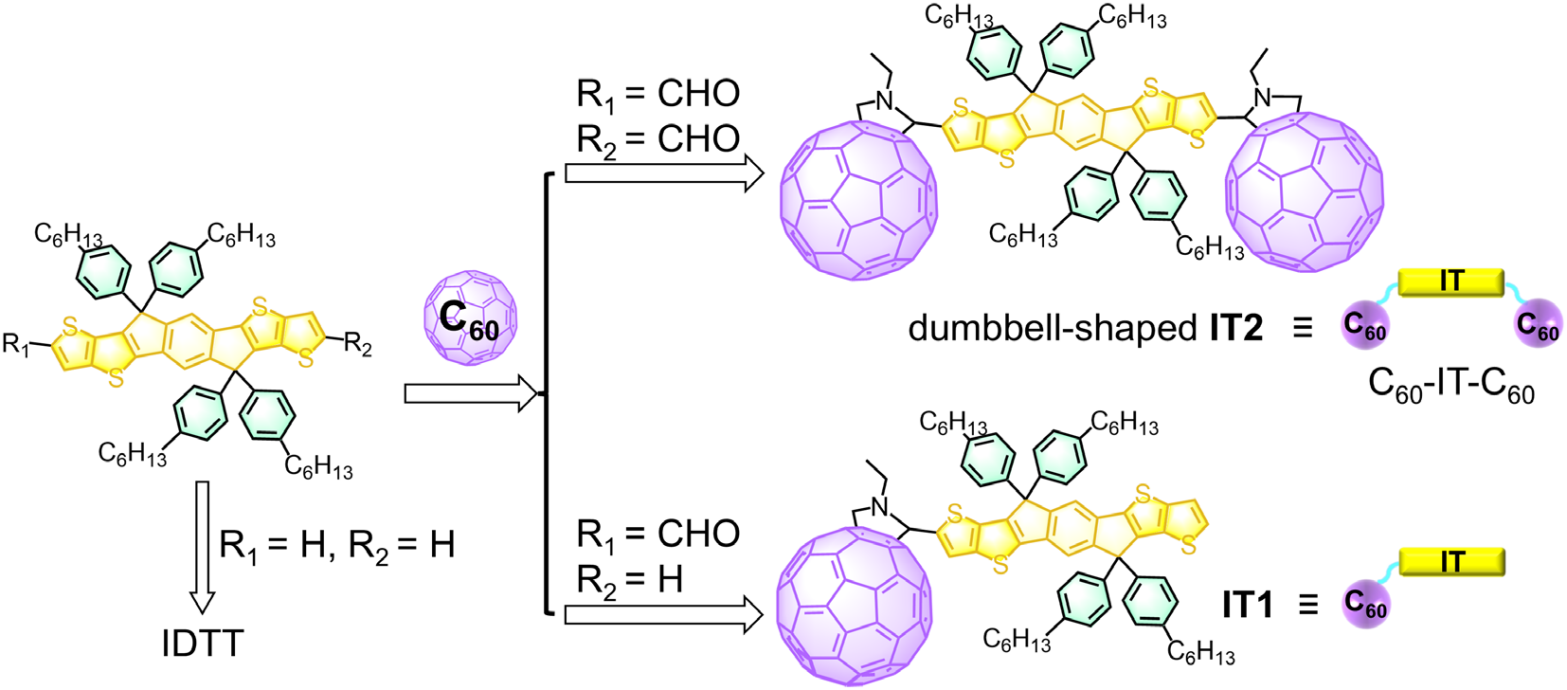
Molecular designs and structures of **IT2**, **IT1**, and the pristine IDTT and C_60_.

In this study, we designed a dumbbell-shaped fullerene-indacenodithiophene triad, **IT2**, with an A-D-A structural arrangement, alongside a single fullerene-modified D-A dyad, **IT1** for comparison (Fig. 1). Fullerenes, represented by C_60_, have well-defined structures and low electronic reorganization energy, making them typical electron acceptors and near-ultraviolet photosensitive molecules (8, 22–25). Their large conjugated structure allows for electronic delocalization, thereby stabilizing them and facilitating the construction of stable and long-lived electron transfer systems (26). Additionally, indacenodithiophene (IDTT) possesses good light-absorption and electron-donating properties (27). Previous research demonstrated that it has two closely spaced oxidation potentials, enabling it to donate two electrons and accumulate two holes (11). Upon selective excitation of C_60_ units in **IT2**, m-ET can occur with a time constant of 0.5 ps, and the product exhibit a lifetime of 10 μs. In contrast, no detectable m-ET product was observed when the donor in **IT2** was excited, or when C_60_ in **IT1** was excited. Importantly, the m-ET rate in **IT2** is 23 times faster than s-ET, and the lifetime of the m-ET product is 1.4 × 10^5^ times longer than that of the s-ET product. These properties can be qualitatively attributed to the larger driving force, lower reorganization energy, and smaller structural changes required for dication generation in **IT2** during m-ET, along with the closed-shell structure of the donor in its dicationic state. This endows **IT2** with superior photocatalytic capability for multielectron oxidation, as demonstrated in experiments on the photocatalytic oxidation of 1,2,3,4-tetrahydroisoquinoline (THIQ) and dithiothreitol (DTE^red^) (7, 28). This work achieved ultrafast m-ET and a long-lived charge-accumulated state in a dumbbell-shaped fullerene triad, providing important insights for the study of m-ET and charge accumulation systems.

## Results

### Synthesis and Characterization.

The double fullerene-modified dumbbell molecule, **IT2** (C_60_-IT-C_60_), and the single fullerene-modified **IT1** were synthesized via 1,3-dipolar cycloaddition (Fig. 1). Detailed synthesis, purification, and characterization steps are listed in *SI Appendix*, Figs. S1–S4. Structurally, the dumbbell-shaped **IT2** can exist in *cis* or *trans* configurations, which may coexist in the products and are challenging to separate and characterize. We optimized both *cis* and *trans* configurations, finding that the *cis*-configuration is energetically favorable, with a minor energy difference (~8.5 meV) suggesting that both configurations are feasible (*SI Appendix*, Fig. S5). Structural diagrams of **IT2** will henceforth be represented using the *cis*-configuration.

### Steady-State Spectra.

**Fig. 2.**
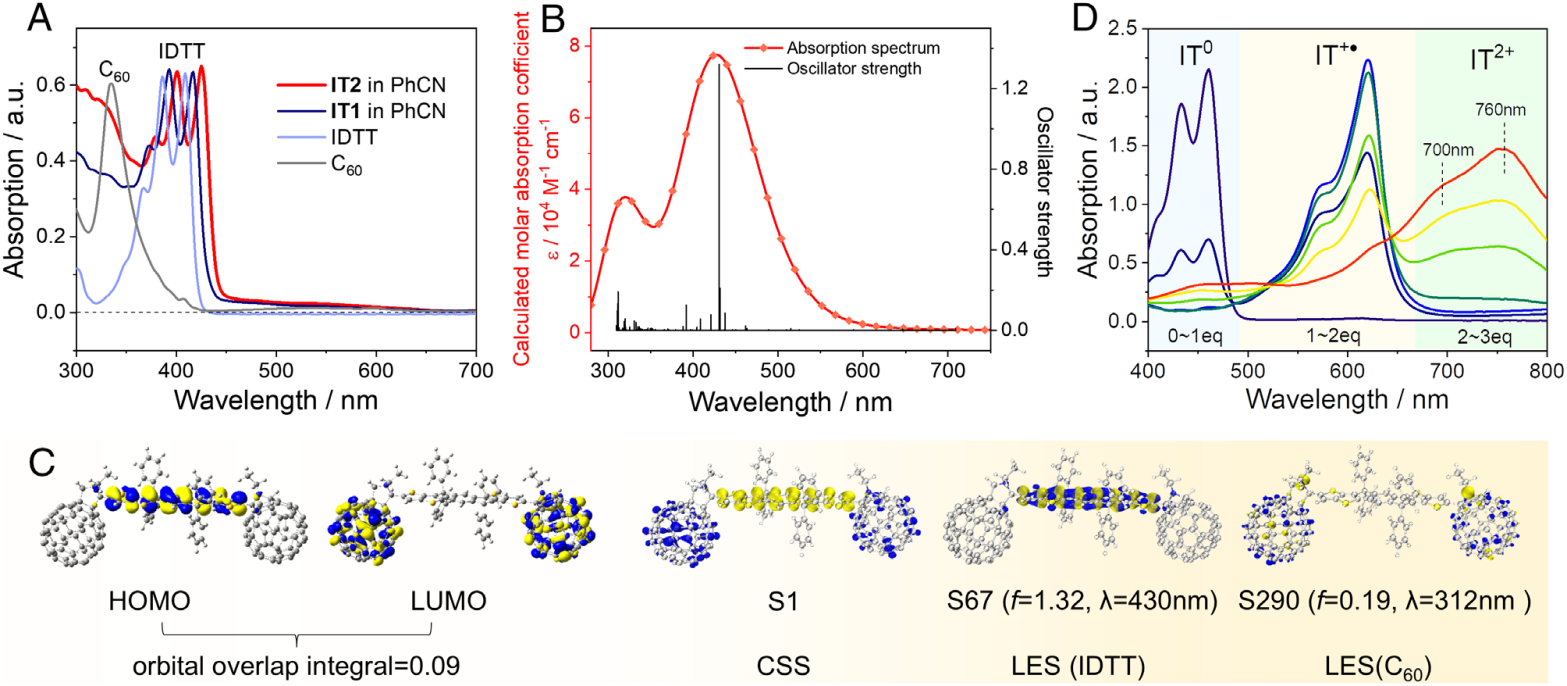
Steady-state absorption spectra and molecular orbital analysis. (*A*) Steady-state absorption spectra of **IT2**, **IT1**, along with the pristine C_60_ and IDTT in PhCN. (*B*) Calculated absorption spectrum and oscillator strength of **IT2** using TD-DFT at B3LYP-D3BJ/6-31G* level. (*C*) Analysis of the orbital interactions, including HOMO and LUMO distributions at ground state (isovalue = 0.02), as well as electron–hole distributions at S1, S67, and S290 excited states (isovalue = 0.001, blue represents electron distribution and yellow represents hole distribution). (*D*) Spectral changes of IDTT-2CHO during chemical oxidation on adding 0 to 3 equivalents (eq) of NOSbF_6_ in CH_2_Cl_2_.

The steady-state absorption spectra of **IT2**, **IT1**, and the pristine molecules IDTT and C_60_ were measured in benzonitrile (PhCN) (Fig. 2*A*). The results show that there is minimal overlap in maximum absorption of C_60_ and IDTT. Combined with the time-dependent density functional theory (TD-DFT) calculations, it can be revealed that the absorption spectrum of **IT2** is a simple superposition of the absorptions of pristine IDTT and C_60_ (Fig. 2*B*), with the S0→S67 transition corresponding to the maximum absorption of IDTT (2.89 eV, oscillator strength, *f* = 1.32), and the S0→S290 transition matched with C_60_ absorption (3.98 eV, *f* = 0.19). Analysis of the electron–hole distribution indicates that both S67 and S290 are localized excited states (LES), allowing for selective excitation of either C_60_ or IDTT without affecting each other (29, 30). Additionally, the HOMO and LUMO of **IT2** exhibit almost no overlap (with an overlap integral of only 0.09) (29), and the holes and electrons in S1 (charge-separated state, CSS) are distributed in the C_60_ and IDTT units, respectively, with no overlap (Fig. 2*C*). Therefore, selective excitation of C_60_ (e.g., at 330 nm) or IDTT (e.g., at 410 nm) can be considered as local excitations, with negligible mutual influence.

To distinguish between photoinduced s-ET or m-ET in **IT2**, nitrosonium hexafluoroantimonate (NOSbF_6_) oxidant was titrated into the IDTT solution in dichloromethane, and changes in the absorption spectra after chemical oxidation were monitored in real-time (Fig. 2*D*). The results demonstrate absorption peaks of the IDTT cation (IT^+•^) at approximately 620 nm, and the IDTT dication (IT^2+^) around 760 nm, providing spectral criteria for assessing photoinduced s-ET or m-ET.

### Electrochemical and Energetic Analysis.

Electrochemical measurements were performed to study the thermodynamic feasibility and energetic relationship between s-ET and m-ET. Cyclic voltammetry (CV) indicated that IDTT exhibited two pairs of reversible redox peaks within the potential window of PhCN electrolyte, corresponding to one- and two-electron oxidation, respectively (*SI Appendix*, Fig. S6 and Table S1). The peak current of the two-electron oxidation peak was higher than that of the one-electron oxidation peak, suggesting that two-electron oxidation of IDTT may occur more readily. Additionally, the potential offset between the first (^1^E_ox_) and second oxidation potentials (^2^E_ox_) is only 0.25 V, making two-electron transfer becoming an exothermic process after excited (15). For the **IT2** triad and **IT1** dyad, the reversibility of the redox potentials decreased, with the peak in the ^1^E_ox_ in IDTT unit almost disappearing and replaced by a more pronounced ^2^E_ox_. The peak current corresponding to the first reduction potential (^1^E_red_) in **IT2** was nearly twice that in **IT1**, reflecting the involvement of two electrons in the reduction process, originating from the two C_60_ units of the dumbbell-shaped molecule (26, 31).

On the basis of the electrochemical results, we estimated the energy relationship between the s-ET and m-ET processes for **IT2** in PhCN (*SI Appendix*, Table S2) (32). The driving force for charge separation (ΔG_CS_) during s-ET is estimated as −0.42 eV, while the ΔG_CS_ for m-ET (transferred two electrons) is approximately −0.59 eV, indicating that both s-ET and m-ET are thermodynamically feasible processes.

### Transient Absorption Investigation.

**Fig. 3.**
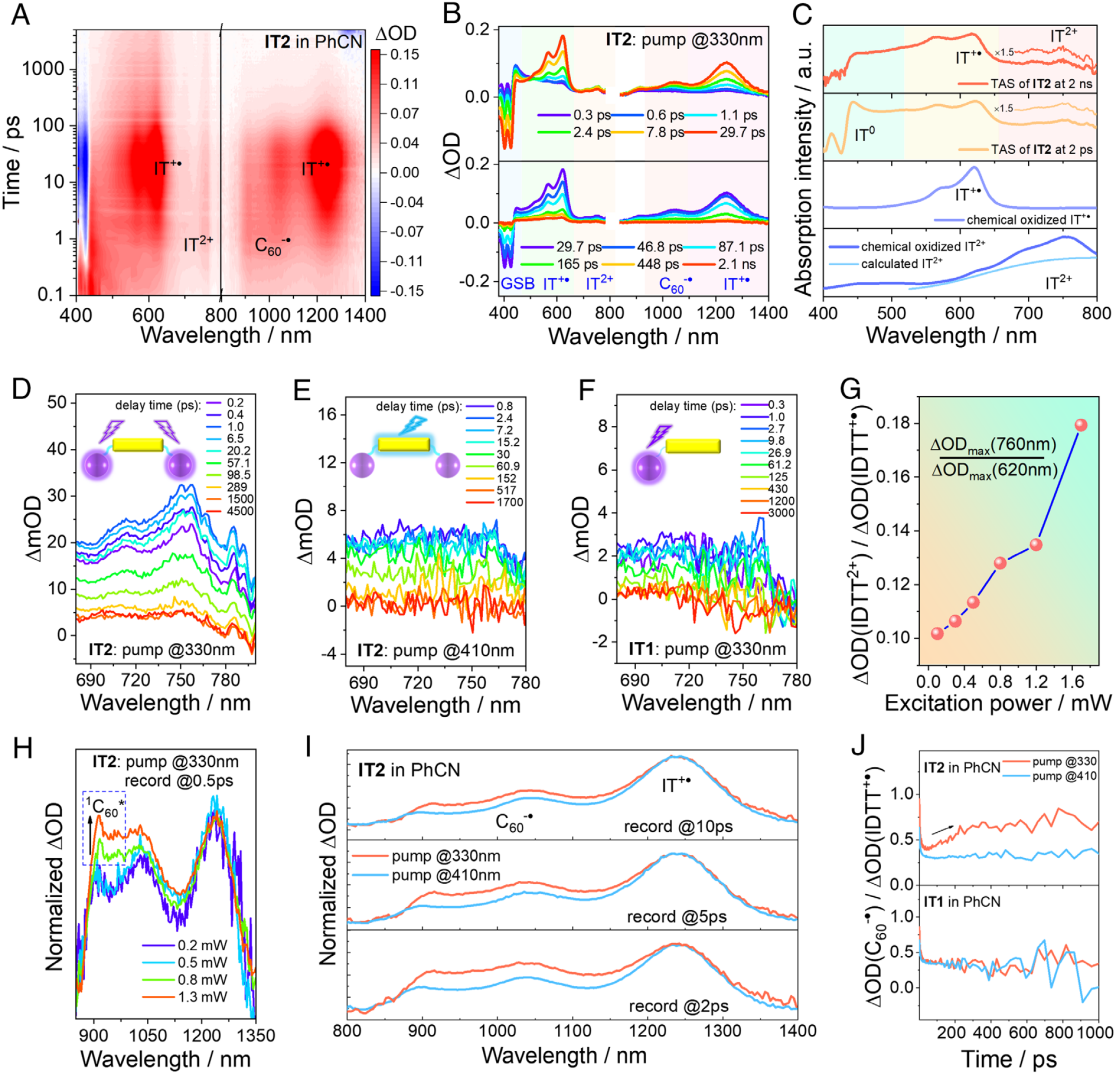
Transient absorption analysis. (*A*) Contour plot of **IT2** in PhCN under 330 nm excitation. (*B*) Selected transient absorption spectra (TAS) of **IT2** in PhCN under 330 nm excitation. (*C*) Comparison of the selected TAS of **IT2** at 2 ps and 2 ns, absorption spectra of the cation and dication upon chemical oxidation, along with calculated dication absorption by TD-DFT at the B3LYP-D3BJ/6-31G* level. (*D*–*F*) Selected TAS around dication absorption of (*D*) **IT2** excited at 330 nm, (*E*) **IT2** excited at 410 nm, and (*F*) **IT1** excited at 330 nm in PhCN under the same excitation power (~0.8 mw). (*G*) Ratio of maximum absorption intensities of the dication and cation for **IT2** excited at 330 nm under various excitation powers. (*H*) Normalized TAS in the NIR region of **IT2** excited at 330 nm and recorded at 0.5 ps under various excitation powers. (*I*) TAS comparison in **IT2** between 330 nm and 410 nm excitation, probed at the NIR region and recorded at 2 ps, 5 ps, and 10 ps. (*J*) Ratio of C_60_^-•^ (probed at 1,010 nm) to IT^+•^ (probed at 1,250 nm) absorption intensities over time in **IT2** (*Top*) and **IT1** (*Bottom*), following 330 nm and 410 nm excitation.

To elucidate the photoexcited electron transfer processes, we systematically designed transient absorption experiments. Upon 330 nm excitation of the C_60_ units in **IT2**, excited-state absorption (ESA) appeared near 440 nm within 1 ps, which can be attributed to the LES absorption of C_60_ (^1^C_60_*, Fig. 3 *A* and *B*) (11). In the subsequent 30 ps, this ESA gradually decayed, accompanied by the appearance of IDTT cation (IT^+•^) absorption nearly 620 nm and the formation of ground-state bleaching (GSB) of IDTT at 420 nm, reflecting a s-ET process. Notably, a weak ESA around 760 nm was observed early, corresponding to the doubly oxidized dication absorption according to the chemical oxidation and TD-DFT calculations (Fig. 3*C*). This indicates that exciting the C_60_ units can cause m-ET, and two holes can accumulate in the donor and generate a dication, IT^2+^. Correspondingly, each C_60_ unit can be occupied by an electron. We denote the m-ET product as C_60_^-•^-IT^2+^-C_60_^-•^. For comparison, when using 410 nm to excite the donor in **IT2**, or 330 nm to excite the single-fullerene modified **IT1** under the same excitation power, no dication ESA was observed (Fig. 3 *D*–*F*). Therefore, only when two C_60_ units were excited in the dumbbell-shaped **IT2** could m-ET occur. At this point, high excitation power can increase the probability of both C_60_ units being excited. With increasing power, the dication absorption gradually became more pronounced than the cation absorption (Fig. 3*G*); meanwhile, ^1^C_60_* features also increased with the excitation power, indicating that the m-ET product originated from the excited state containing two ^1^C_60_*, denoted as ^1^C_60_*-IT-^1^C_60_* (Fig. 3*H* and *SI Appendix*, Fig. S7).

Next, we focused on the C_60_ anion (C_60_^-•^) evolution process. In the near-infrared (NIR) region, the ESA around 1,030 nm overlaps with C_60_^-•^ (1,010 nm) and partial IT^+•^ absorption, with 1,250 nm corresponding to IT^+•^ absorption (11, 22). For **IT2**, compared to the 410 nm excitation, the C_60_^-•^ absorption under 330 nm excitation within 10 ps was more significant, and the ratio of C_60_^-•^ to IT^+•^ absorption was larger (Fig. 3 *H* and *I*). This is because 330 nm excitation can cause m-ET, generating two C_60_^-•^ in one **IT2** triad, whereas only s-ET occurs under 410 nm excitation and produces one C_60_^-•^. Furthermore, exciting C_60_ units at 330 nm in **IT2** causes the ratio of C_60_^-•^ to IT^+•^ absorption gradually increased over time, reflecting that C_60_^-•^ decayed more slowly than IT^+•^, while in the case of s-ET (exciting IDTT at 410 nm), this ratio remained nearly constant. For comparison, this ratio in **IT1** was almost identical at either 330 or 410 nm excitation and did not change over time. Thus, selective excitation of C_60_ units in dumbbell-shaped **IT2** can induce m-ET.

**Fig. 4.**
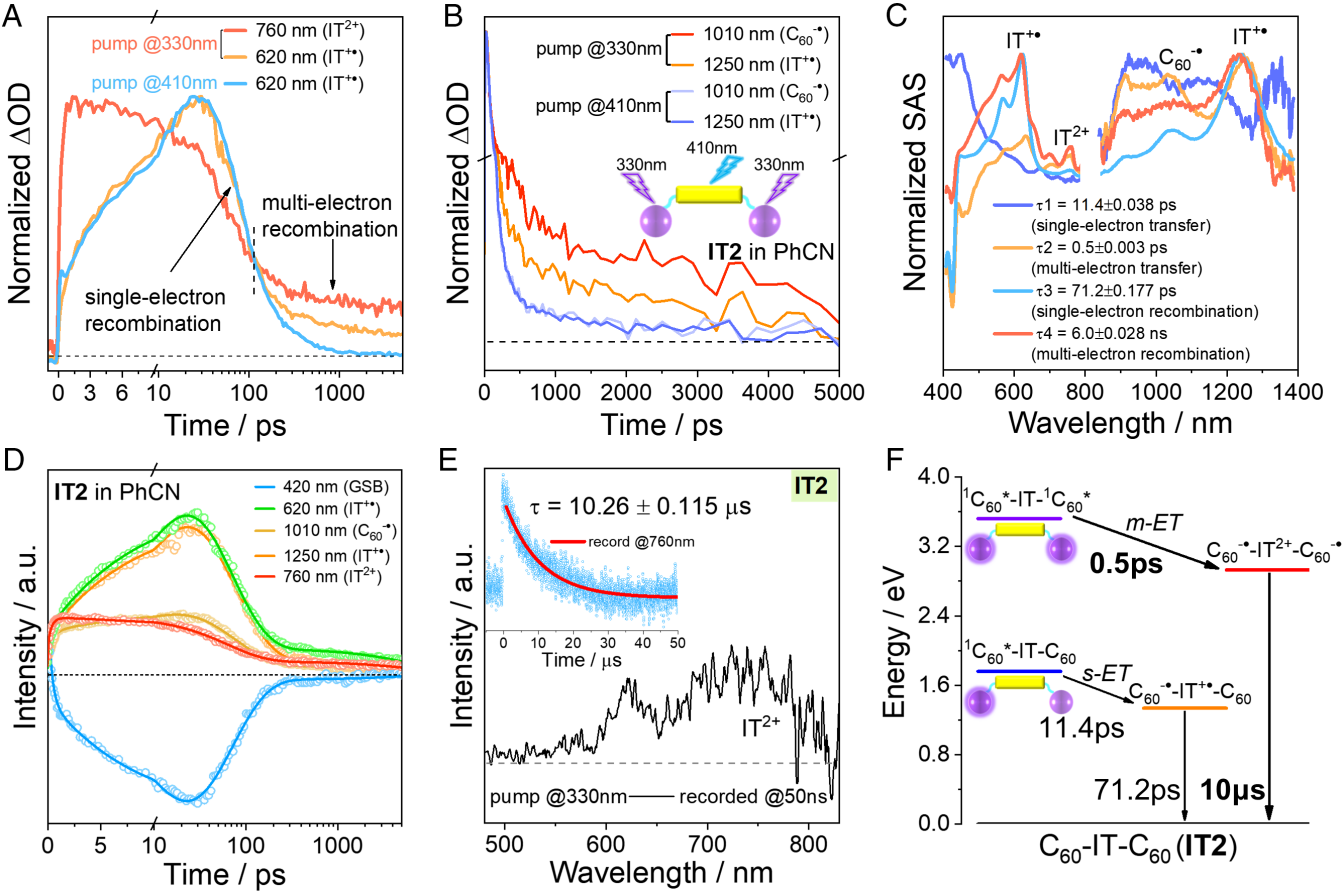
Electron transfer kinetics of **IT2** in PhCN. (*A*) Electron transfer kinetics obtained from femtosecond transient absorption (fs-TA) under 330 nm or 410 nm excitation, probed at 620 nm for s-ET, i.e., cation (IT^+•^) absorption, and at 760 for m-ET, i.e., dication (IT^2+^) absorption. (*B*) Electron transfer kinetics under 330 nm or 410 nm excitation obtained from fs-TA, probed at 1,010 nm for C_60_^-•^ absorption, and at 1,250 nm for IT^+•^ absorption. (*C*) Normalized species-associated spectra (SAS) obtained from target analysis. The time constants and fitting errors for the s-ET/recombination and m-ET/recombination processes are listed here. (*D*) Kinetics of s-ET (probed at 620, 1,010, and 1,250 nm), m-ET (probed at 760 and 1,010 nm), and GSB (probed at 420 nm), along with the fitted results obtained from target analysis following 330 nm excitation. (*E*) ns-TA spectrum of **IT2** in PhCN recorded at 50 ns following 330 nm excitation. Inset: Dication decay kinetics probed at 760 nm. Using single-exponential fitting, the dication lifetime was determined to be 10 μs. (*F*) Jablonski diagram of the electron transfer processes at the excited states. The energy levels were estimated from the electrochemistry results, and the corresponding time constants for electron transfer were obtained from the target analysis.

To further explore the mechanisms of s-ET and m-ET, we conducted a comprehensive analysis of the excited-state kinetics. I) CS. For both the 330 and 410 nm excitations of **IT2**, the cation generation kinetics (probed at 620 nm) were almost identical, indicating that the s-ET process was excited-wavelength-independent (Fig. 4*A*). Interestingly, following 330 nm excitation, the dication absorption at 760 nm reached its maximum intensity within 2 ps and then decayed slowly, whereas the cation slowly reached its maximum intensity within 30 ps. Meanwhile, compared to the cation kinetics in the NIR region, C_60_^-•^ generation exhibited two processes, and the proportion of the fast-generated component increased with increasing excitation power (*SI Appendix*, Fig. S8). These indicate that m-ET occurs faster than s-ET. II) CR. For both 330 and 410 nm excitations, the CR kinetics probed at 620 nm for the cation were almost identical within 100 ps, reflecting a single-electron recombination process (because there is no m-ET feature when excited at 410 nm). However, a slower decay process existed in **IT2** after 100 ps under 330 nm excitation and did not deactivate completely within 5 ns, which was absent in the s-ET product under 410 nm excitation (Fig. 4*A*). This is similar to the dication decay process (around 760 nm) after 100 ps. Additionally, C_60_^-•^ decayed more slowly than IT^+•^ under 330 nm excitation, while they were synchronous in the s-ET product under 410 nm excitation (Fig. 4*B*). These results manifested that the slow recombination process originated from the decay of the m-ET product, which had a longer lifetime than that of the s-ET product.

Based on transient absorption spectra (TAS) and kinetic analysis, the s-ET and m-ET are relatively independent (parallel) processes. Therefore, we employed target analysis to fit the kinetics and obtained species-associated spectra (SAS, Fig. 4*C*). The evolution processes of the first and third components correspond to s-ET and recombination, while the second and fourth components’ evolution processes correspond to m-ET and recombination, characterized by pronounced IT^2+^ absorption features and stronger C_60_^-•^ absorption. Several selected kinetic curves at characteristic wavelengths demonstrate consistency between the fitting results and the original data (Fig. 4*D*). Accordingly, the time constant for s-ET is 11.4 ps, whereas for m-ET, it is only 0.5 ps, indicating a remarkably faster m-ET process than s-ET (1, 15, 16, 33). Additionally, the m-ET product exhibits a long lifetime exceeding 6 ns when excited at 330 nm, whereas the s-ET product lifetime is only 71.2 ps, consistent with the lifetimes of **IT2** excited at 410 nm, and **IT1** excited at 330 nm (*SI Appendix*, Fig. S9). Employing nanosecond transient absorption (ns-TA) with 330 nm excitation, a prominent ESA around 760 nm was observed. Distinct from the localized triplet state of C_60_ (^3^C_60_*), this can be attributed to the absorption of the m-ET product IT^2+^ (*SI Appendix*, Fig. S10). Using single-exponential fitting, the m-ET products lifetime reaches an unexpected 10 μs (Fig. 4*E*). CS and CR time constants during s-ET or m-ET for **IT2** and **IT1** are summarized in *SI Appendix*, Table S3. Combining this with the analysis of excited-state energy levels, we proposed the photophysical processes of **IT2** under 330 nm excitation (Fig. 4*F*). It is evident that m-ET and s-ET are relatively independent processes, with m-ET occurring faster than s-ET, indicating that the m-ET process does not rely on the s-ET product. Additionally, due to the instrument time resolution constraints, stepwise processes of m-ET cannot be excluded, for instance, from the initial-state ^1^C_60_*-IT-^1^C_60_* to the intermediate-state C_60_^-•^-IT^+•^-^1^C_60_* (rate constant, k_1_), and then to the final charge-accumulated state C_60_^-•^-IT^2+^-C_60_^-•^ (rate constant, k_2_). As the absorption spectrum of the intermediate product is not resolved in transient absorption, this indicates k_2_ >> k_1_. Under these conditions, the fitted rate constant (0.5 ps)^−1^ can be considered approximately the total rate constant for the entire two-electron transfer process from ^1^C_60_*-IT-^1^C_60_* to C_60_^-•^-IT^2+^-C_60_^-•^ (see *SI Appendix* for details). Impressively, in the single-molecule **IT2**, the rate of m-ET is 23 times faster than that of s-ET, and the m-ET product lifetime is 1.4 × 10^5^ times longer than the s-ET product.

### Mechanism Explanation of the m-ET.

**Fig. 5.**
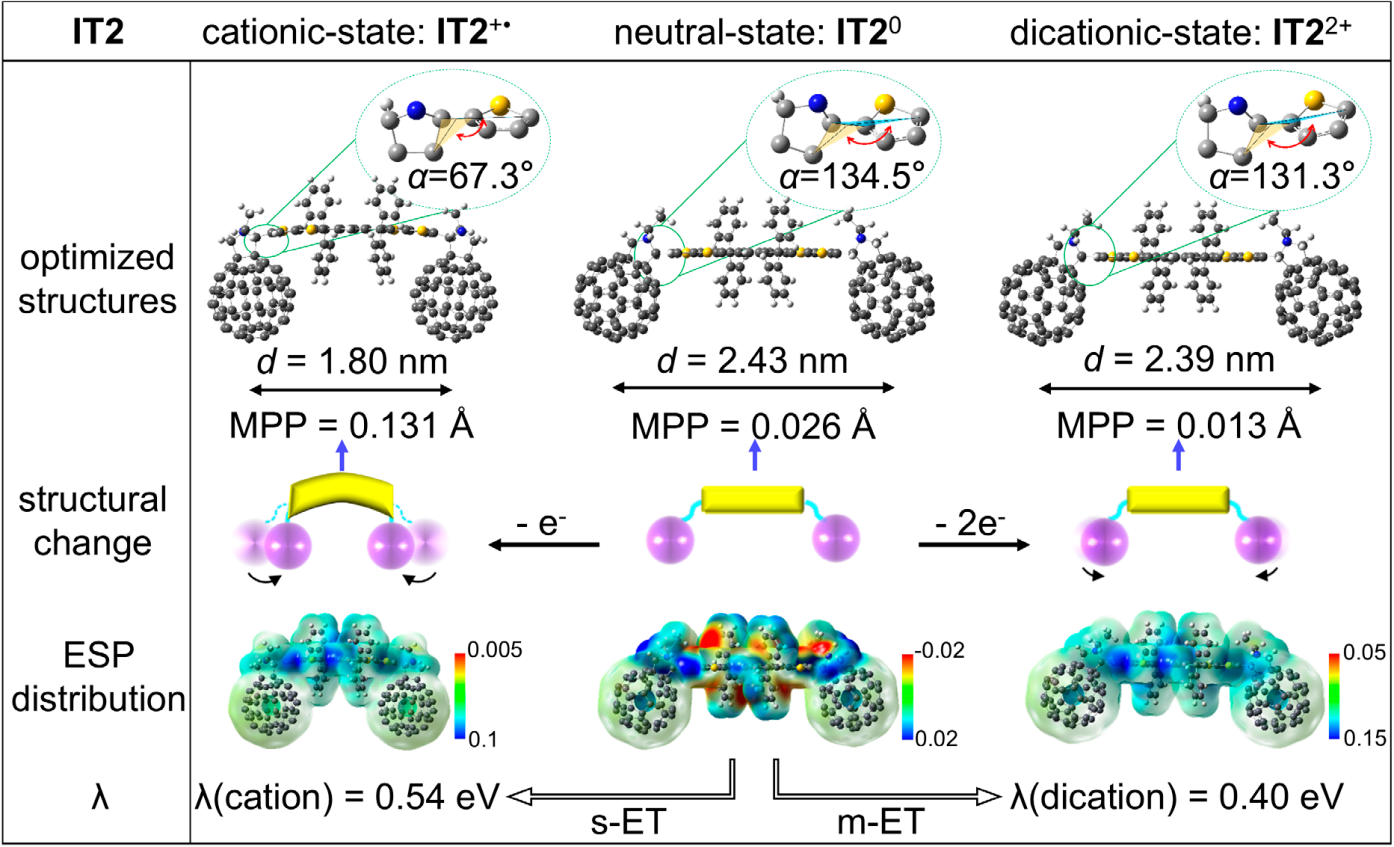
Computational analysis. Computational results including optimized structures of **IT2** at neutral (**IT2^0^**), cationic (**IT2^+•^**), and dicationic (**IT2^2+^**) states (first row), a schematic diagram of structural changes when losing one or two electrons (second row) from **IT2**, the ESP distribution (third row), and the hole-transfer reorganization energy (λ) during s-ET and m-ET processes. The calculated values of centroid distance (*d*), dihedral angle (*α*), and MPP are also shown here. All the computational results are calculated by DFT at the B3LYP-D3BJ/6-31G* level.

The unexpected ultrafast m-ET and long-lived m-ET products were simultaneously obtained in single-molecule **IT2**, which can occur across various solvents including chlorobenzene, *o*-dichlorobenzene, dichloromethane, chloroform, and benzonitrile (*SI Appendix*, Fig. S11). To examine the mechanism, we integrated electrochemical studies and quantum chemical calculations to analyze the m-ET process from the perspectives of the driving force, reorganization energy, and structural changes (Fig. 5). The optimized atomic coordinates and corresponding Gibbs free energies for the neutral, cationic, and dicationic states of **IT2** are provided in *SI Appendix*, Tables S4–S6. First, based on electrochemical analysis, during the m-ET process, the driving force is about −0.59 eV for CS and −2.93 eV for CR; whereas the driving force is −0.42 eV for CS and −1.34 eV for CR during s-ET process (32). Using DFT calculations by Gaussian 16 package at the B3LYP-D3BJ/6-31G* level (29, 34, 35), the reorganization energy for hole transfer for the s-ET process is 0.54 eV, whereas for the m-ET process it is only 0.40 eV. The solvent reorganization energies were left out because s-ET and m-ET both occurred in the same molecular system and solvent. Unfortunately, the electronic coupling for the m-ET process was not obtained because of the unknown geometric structure and wavefunction of ^1^C_60_*-IT-^1^C_60_*. Nevertheless, analysis of the current results can still support the experimental findings. Specifically, during the m-ET process, the driving forces for CS and CR were greater, and the reorganization energy was lower. According to Marcus theory (36), this favors fast electron transfer and slow CR (*SI Appendix*, Fig. S12) (21, 37). Additionally, the electrostatic potential (ESP) distribution shows that the positive charges of cation and dication are nearly delocalized over the donor framework. The two holes accumulated in the conjugated backbone of the donor tended to pair to reduce the system energy, and the formed closed-shell structure stabilized the m-ET product (38). Furthermore, structural optimization shows that in the cationic state of **IT2**, the distortion of the IDTT framework is quite significant due to its open-shell structure (39). The two carbon cages approach closer together, with the centroid distance (*d*) shortening from 2.43 nm to 1.80 nm, the dihedral angle (*α*) decreasing from 134.5° to 67.3°, and the molecular planarity parameter (MPP) increasing from 0.026 Å to 0.131 Å (29, 40), distinctly different from the neutral-state. For comparison, the dicationic **IT2** structure is almost identical to the neutral state due to its closed-shell structure (Fig. 5). In **IT2**, the donor losing two electrons cause smaller structural changes than losing one electron, which can also enhance the thermodynamic and kinetic advantages of the m-ET process, and result in fast m-ET and longer lifetimes of the m-ET product than those of s-ET.

Here, we qualitatively reveal the reasons for the unique kinetic characteristics of m-ET, which benefit from the greater driving force, lower reorganization energy, and smaller structural changes. More importantly, this makes the m-ET products easier to form than the s-ET products, which contributes to the fact that m-ET and s-ET are two parallel processes in **IT2**, rather than sequential processes of electron transfer from the s-ET product (C_60_^-•^-IT^+•^-C_60_) to the m-ET product (C_60_^-•^-IT^2+^-C_60_^-•^). This makes it possible for m-ET to be faster, and its product has a longer lifetime than those of s-ET. It is foreseeable that electronic coupling in the m-ET process can also play an important role (41). If the structural characteristics and wavefunction information of the doubly singlet excited state and charge-accumulated state are known, a deeper kinetic mechanism of ultrafast m-ET may be uncovered. Our further research will be dedicated to exploring the m-ET mechanism toward these aspects.

### Application of the m-ET.

**Fig. 6.**
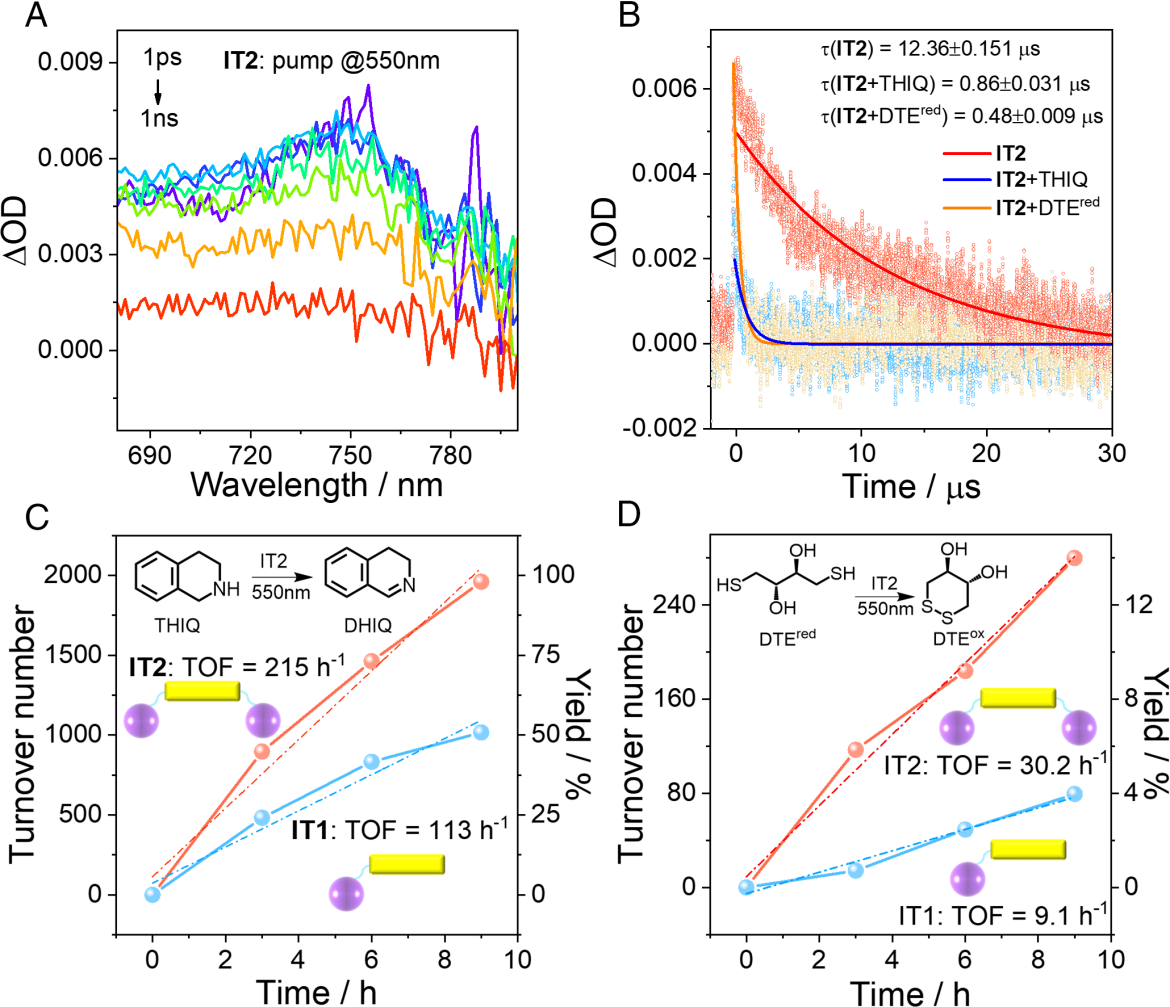
Photocatalytic performance of **IT2**. (*A*) Selected TAS around dication absorption of **IT2** in PhCN following 550 nm excitation. (*B*) Decay kinetics of dication (probed at 760 nm) in **IT2**, **IT2** mixed with a 1,000-fold excess of THIQ, and **IT2** mixed with a 1,000-fold excess of DTE^red^ following 532 nm excitation. (*C*) Photocatalytic turnover numbers (TON) of THIQ and product yields of DHIQ for **IT2** compared with **IT1** at various 550 nm LED irradiation times. Structures of THIQ and DHIQ are provided, along with calculated turnover frequency (TOF). (*D*) Photocatalytic TON of DTE^red^ and product yields of DTE^ox^ for **IT2** compared with **IT1** at various 550 nm LED irradiation times. Structures of DTE^red^ and DTE^ox^ are provided, along with calculated TOF. Yield was determined by standard curve using chromatography pure dichloromethane as an internal standard.

The ability to accumulated multiple electrons markedly enhances the application value of **IT2**. For example, charge accumulation via m-ET endows **IT2** with enhanced performance in photocatalyzing multielectron-involved oxidation reactions. Generally, completing multielectron reactions in s-ET systems requires the participation of multiple molecules, limiting the reaction efficiency; however, this challenge can be mitigated in m-ET systems (7, 19). We studied the photocatalytic oxidation of THIQ to 3,4-dihydroisoquinoline (DHIQ), and the oxidation of dithioerythritol (DTE^red^) to 4,5-dihydroxy-1,2-dithiane (DTE^ox^) in PhCN, both of which involve two electrons participation (28), and chose **IT2** or **IT1** in a one-thousandth equivalent as the photocatalyst (*SI Appendix*). To avoid potential damage to the reaction systems by ultraviolet (UV) light, a 550 nm light-emitting diode (LED) was chosen as the light source, at which wavelength IDTT has almost no absorption, while C_60_ can still be excited (Fig. 2*A*), and m-ET can also occur in **IT2** proved by the TAS (Fig. 6*A*). Employing ns-TA following 532 nm excitation of **IT2**, it can be observed that when containing photocatalytic substrates, the decay rate of the dication IT^2+^ around 760 nm significantly accelerated (with the lifetime of 0.86 μs mixed with THIQ, and 0.48 μs mixed with DTE^red^), indicating that in photocatalytic multielectron oxidation reactions, the dication was effectively quenched by THIQ or DTE^red^ (Fig. 6*B* and *SI Appendix*, Figs. S13 and S14) (42). ^1^H NMR analysis shows that when **IT2** was used as the photocatalyst, the yield of DHIQ exceeded 98 % after 9 h of 550 nm illumination, with a catalytic turnover frequency (TOF) of 215 h^−1^; while **IT1** resulted in the yield of DHIQ only 50.8 %, with a TOF of 113 h^−1^ (Fig. 6*C* and *SI Appendix*, Figs. S15, S16, S19, and S20). No DHIQ product was detected after 9 h of 550 nm LED irradiation of THIQ in the absence of a photocatalyst. And the yield of DTE^ox^ was 14 % with **IT2** as the photocatalyst (TOF = 30.2 h^−1^), whereas with **IT1** as the photocatalyst, the yield of DTE^ox^ was only 4.0 % (TOF = 9.1 h^−1^) (Fig. 6*D* and *SI Appendix*, Figs. S17, S18, S21, and S22) (7, 19). Therefore, the long-lived m-ET product in **IT2** exhibits superior photocatalytic capability.

## Discussion

In summary, an A-D-A type dumbbell-shaped fullerene triad, **IT2**, was successfully constructed for m-ET and charge accumulation within a single molecule. Exciting C_60_ units can induce ultrafast m-ET with a time constant of 0.5 ps, and generate a long-lived charge-accumulated state lasting 10 μs. In comparison, exciting the donor IDTT in the **IT2** triad, or exciting the C_60_ in a single fullerene-modified **IT1** dyad, resulted in s-ET only. Kinetic analysis showed that the m-ET rate was 23 times faster than that of s-ET, and the lifetime of the generated charge-accumulated state was 1.4 × 10^5^ times longer than the s-ET product. These benefits stemmed from the larger driving force, lower reorganization energy, and smaller structural changes during m-ET in dumbbell-shaped **IT2**, along with the closed shell of the donor unit in m-ET product. The m-ET and charge accumulation features allowed **IT2** to better facilitate multielectron-involved redox reactions. In photocatalytic oxidation of THIQ and dithioerythritol (DTE^red^), the catalytic efficiency of **IT2** was 2 to 3 times higher than **IT1**.

This work represents achievement of faster m-ET than s-ET in a single molecule, along with obtaining a m-ET product with a longer lifetime than the s-ET product. This advancement significantly contributes to the study and application of m-ET systems. Beyond photochemical reactions, it holds enormous potential for single-molecule devices with rapid charge accumulation rates, promising rapid intramolecular electron and information storage (43). Furthermore, further studies on the structures and wavefunction characteristics of the doubly singlet-state or charge-accumulated state could potentially provide deeper insights into m-ET mechanisms.

## Materials and Methods

### Materials.

The IDTT precursors, IDTT-2CHO and IDTT-CHO were purchased from *Organtec*, *Ltd*. The dumbbell-shaped molecule **IT2** and its counterpart **IT1** were synthesized in toluene solution via the prato reaction in the presence of *N*-ethylglycine and fullerene C_60_ (44). The detailed synthetic methodologies are illustrated in *SI Appendix*.

### Purification and Characterization.

The crude **IT2** and **IT1** products were separated using a silica column (eluent: petroleum ether and toluene 4:1) and further purified by high-performance liquid chromatography using a Buckprep column (10 mm × 250 mm) with toluene as the eluent at a flow rate of 6 mL/min. ^1^H NMR was carried out in CDCl_3_ on an Avance-400 (400 MHz) spectrometer (Bruker) at 298 K with chemical shifts (δ, ppm) reported relative to the solvent peak. Mass spectrometry was performed using a matrix-assisted laser desorption/ionization fourier-transform ion cyclotron resonance mass spectrometer (MALDI-FT-ICR-MS) spectrometer (Solarix, Bruker).

### Steady-State Spectra.

The steady-state absorption spectra were recorded using a UH4150 spectrophotometer (HITACHI). The sample concentration for steady-state spectra measurement was approximately 2 × 10^−5^ M.

### Electrochemical Measurement.

CV measurements were carried out by a CHI760E electrochemical analyzer (Chenhua, Shanghai) in 0.05 M tetrabutylammonium hexafluorophosphate in deaerated benzonitrile (PhCN) with the scan rate of 0.1 V s^−1^. A three-electrode configuration was employed. Specifically, a glassy carbon electrode was used as the working electrode, a platinum wire as the counter electrode, and a saturated Ag/AgCl electrode as the reference electrode. The sample concentration was about 10^−4^ M. All results were corrected using a ferrocene/ferrocenium couple (Fc/Fc^+^).

### Transient Absorption Measurement.

Femtosecond transient absorption (fs-TA) (Ultrafast System, Helios) measurements were performed at the Institute of Physics and Chemistry, Chinese Academy of Sciences. A similar description of the experimental methods can be found elsewhere (45). Briefly, a femtosecond laser (Coherent Inc.) delivered 25 fs pulses at 1 kHz, and the output was split to generate a white-light continuum. The excitation wavelength was obtained using tunable optical parametric amplifiers (TOPAS-C; Light Conversion). The specific excitation wavelengths and densities are described in the main text. The continuum was used as a broadband optical probe from the near-UV to near-IR regions. The probe from 350 to 750 nm was generated by focusing the fundamental laser beam onto a 3 mm CaF_2_ plate, which was oriented and continuously shifted in perpendicular directions. A near-IR probe was generated by focusing the beam on the YAG crystal. The TA spectrum was calculated from consecutive pump-on and pump-off measurements and averaged over 400 shots. The SAS were obtained using GloTarAn, a program based on the R package TIMP and singular value decomposition (46, 47).

ns-TA experiment was carried out by a commercial nanosecond laser flash photolysis spectrometer (LP980-KS, Edinburgh Instruments Ltd., Livingston, UK). A similar description of the experimental methods can be found elsewhere (48). Briefly, the pump laser pulses were generated from the Optical Parametric Oscillator (PrimoScan ULD400, Spectra-Physics, US) at 330 nm, with the full width at half maximum around 10 ns. The probe light was provided by a 150 W pulsed xenon arc lamp. The transmitted probe light was measured either by a single PMT detector (Hamamatsu R928), using a Tektronix Model MDO3052 (100 MHz, 1.25 GS s^−1^) digital oscilloscope, at a specified wavelength for kinetic analysis or by an ICCD camera (DH320T, Andor, UK) for spectral analysis.

All the fs-TA measurements are carried out in a quartz cuvette with a 1 mm optical path, with sample concentrations of approximately 10^−4^ M. The ns-TA experiments are performed in a quartz cuvette with a 10 mm optical path, and the sample concentrations were approximately 6 × 10^−5^ M. Before and after each measurement, steady-state absorption spectra were employed to ensure that no obvious photodegradation occurred during the TA measurement.

### Quantum Chemical Calculation.

The structures of **IT2** and **IT1** were optimized using the Gaussian 16 package at the B3LYP-D3BJ/6-31G* level (34, 35). To simplify the calculation, the side chain was replaced by benzene (49). And the Multiwfn program was used to calculate the absorption spectra and molecular orbitals (29). The atomic coordinates of all optimized structures and their Gibbs free energies are provided in *SI Appendix*.

### Photocatalytic Reaction.

Photocatalytic oxidation was carried out at room temperature in PhCN in an optical reactor with a 550 nm LED as the light source, and the ^1^H NMR spectroscopy was used to monitor the reaction process and perform quantitative analysis.

## Supplementary Material

Appendix 01 (PDF)

## Data Availability

All study data are included in the article and/or *SI Appendix*.
