# Supplementary material for: Photoinduced ultrafast multielectron transfer and long-lived charge-accumulated state in a fullerene-indacenodithiophene dumbbell triad
Source: Proc Natl Acad Sci U S A. 2024 Dec 5;121(50):e2414671121. doi: 10.1073/pnas.2414671121 (PMC11648911; doi:10.1073/pnas.2414671121)
Supplement: Supplementary file 1 — Appendix 01 (PDF) [file pnas.2414671121.sapp.pdf]

PNAS

Supporting Information for

Photoinduced ultrafast multielectron transfer and long-lived charge-accumulated state in a fullerene-indacenodithiophene dumbbell triad

Chong Wang[a,b], Bo Wu[a,b]*, Yang Li[c], Ying Jiang[a,b], Tianyang Dong[a,b], Shen Zhou[d], Chunru Wang[a,b]*, and Chunli Bai[a,b]*

[a] Beijing National Laboratory for Molecular Sciences, Key Laboratory of Molecular Nanostructure and Nanotechnology, Institute of Chemistry, Chinese Academy of Sciences, Beijing 100190, China

[b] University of Chinese Academy of Sciences, Beijing 100049, China

[c] School of Science, Beijing University of Posts and Telecommunications (BUPT), Beijing 100876, China

[d] College of Science, Hunan Key Laboratory of Mechanism and Technology of Quantum Information, National University of Defense Technology, Changsha 410003, China

* Corresponding authors: Bo Wu, Chunru Wang, and Chunli Bai.

Email: zkywubo@iccas.ac.cn; crwang@iccas.ac.cn; clbai@cas.cn

This PDF file includes:

Supporting text
Figures S1 to S22
Tables S1 to S6
SI References

Supporting Information Text

Synthesis of IT2 ($C_{196}H_{88}N_2S_4$).

The indacenodithiophene precursors are purchased from *Organtec Ltd*. The A-D-A structural and dumbbell-shaped fullerene-indacenodithiophene triad (**IT2**) was synthesized by prato reaction (*1*), as shown in **Fig. S1**. Briefly, 510.00 mg IDTT-2CHO (0.474 mmol, 1 equiv), 1002.25 mg C_{60} (1.39 mmol, 2.9 equiv) and 145.69 mg *N*-ethylglycine (1.41 mmol, 2.9 equiv) were dissolved in 300 mL toluene. Under N_2 protection, the reactants mixture was heated to 130 ^{o}C, and then refluxed for 12 h at this temperature. When the reaction completed, the solvent was evaporated under reduced pressure, then the crude product was separated by silica gel column chromatography (eluent; petroleum ether and toluene 4:1). During this separation process, unreacted C_{60} elutes first, followed by the second fraction, which is the target product, the bis-fullerene substituted dumbbell-shaped **IT2**. The third fraction to elute is a derivative with a single fullerene modification, containing an unreacted aldehyde group on the donor framework. The raw product **IT2** can be further purified by HPLC with buckprep column (toluene, 6 mL/min). ^{1}H NMR ($CDCl_3$, 400 MHz, δ/ppm): δ 7.71 (s, 2H), δ 7.46 (s, 2H), δ 7.13-6.91 (m, 16H), δ 5.49 (s, 2H), δ 5.17 (s, 2H), δ 4.30 (m, 2H), δ 3.69 (s, 2H), δ 2.88 (s, 2H), δ 2.53-2.42 (m, 8H), δ 1.29-1.23 (m, 32H), δ 0.90-0.83 (m, 18H). Molecular weight (m/z): 2958.593.

Synthesis of IT1.

The synthesis method (**Fig. S1**), purification, and characterization results of **IT1** have been reported in our previous work (*2*).

Comparison of the *trans-* and *cis*-configuration of the IT2.

The two fullerenes in **IT2** can be on the same side of the donor backbone (*cis*-configuration), or on opposite sides (*trans*-configuration); additionally, the different orientations of the fullerene pyrrole-rings can lead to different geometric configurations of the **IT2** triad (*3, 4*). Here the influence of the latter on the electronic effects of the **IT2** triad can be ignored. We only consider the *cis*- and *trans*-configurations of the dumbbell-shaped **IT2**. These two configurations may coexist, as the ^{1}H-NMR spectrum of **IT2** indicated that the protons on the two pyrrole-rings of the fullerenes were essentially in a symmetric chemical environment, making them difficult to separate individually. Herein, these two configurations were optimized using DFT at the B3LYP-D3BJ/6-31G* level. The optimized structures are shown in **Fig. S5**, wherein the *cis*-configuration has a lower relative energy, denoted as 0 eV, whereas the relative energy of the *trans*-configuration is slightly higher by 8.5 meV than the *cis*-configuration, indicating that the *cis*-configuration is likely more stable. However, the energy difference between the *cis*- and *trans*-configuration is not significant, indicating that both configurations can exist in **IT2**. The schematic diagrams of the dumbbell-shaped **IT2** involved in the main text all are tentatively described by the *cis*-configuration.

Chemical oxidation of the IDTT-2CHO.

Due to the strong oxidizing nature of nitrosonium hexafluoroantimonate ($NOSbF_6$), the solvent benzonitrile can be oxidized by it. Therefore, the chemical oxidation reaction here is carried out in dichloromethane, with $NOSbF_6$ pre-dissolved in a small amount of acetonitrile.

Estimation of the energies at excited states.

According to the Rehm-Weller equation (*5*), the energy levels of the excited states for **IT2** following excitation can be estimated on the basis of the redox potentials obtained from electrochemistry results (*6*). The terms of Coulomb interactions and solvent reorganization energies included in the equation were left out here, because the overall charge number remains constant during charge transfer reactions, and the measurements were carried out in high-polarity PhCN (*7*).

(1) C_{60}-IT-C_{60}. The ground state energy level of the **IT2**, marked as C_{60}-IT-C_{60} here, is set to 0 eV;

(2) $^1C_{60}$*-IT-C_{60}. When a single C_{60} unit is excited, its energy level corresponds to the energy of the lowest singlet excited state of C_{60}, which is 1.76 eV (*8*);

(3) $C_{60}^{-\bullet}$-$IT^{+\bullet}$-C_{60}. When single electron transfer (s-ET) occurs, the energy level of the product can be estimated by the following formula: $E(C_{60}^{-\bullet}\text{-}IT^{+\bullet}\text{-}C_{60}) = e({}^1E_{ox}(IDTT) - {}^1E_{red}(C_{60})) = 1.34$ eV;

(4) $^1C_{60}$*-IT-$^1C_{60}$*. When both C_{60} units are excited, the energy level corresponds to twice the energy of the lowest singlet excited state of C_{60}, which is 3.52 eV (*6*);

(5) $C_{60}^{-\bullet}$-IT^{2+}-$C_{60}^{-\bullet}$. When double electron transfer (m-ET) occurs, the product energy level can be estimated by the following formula: $E(C_{60}^{-\bullet}\text{-}IT^{2+}\text{-}C_{60}^{-\bullet}) = e({}^1E_{ox}(IDTT) - {}^1E_{red}(C_{60})) + e({}^2E_{ox}(IDTT) - {}^1E_{red}(C_{60})) = 2.93$ eV.

Herein, e is the elementary charge, $^1E_{ox}$(IDTT) is the first oxidation potential of IDTT, $^2E_{ox}$(IDTT) is the second oxidation potential of IDTT, and $^1E_{red}(C_{60})$ is the first reduction potential of C_{60}. These potentials were measured in PhCN electrolyte containing 0.05 M $TBAPF_6$, and corrected using Fc/Fc^+. Accordingly, the driving force for the s-ET process is $\Delta G_{CS(s\text{-}ET)} = E(C_{60}^{-\bullet}\text{-}IT^{+\bullet}\text{-}C_{60}) - E({}^1C_{60}^*\text{-}IT\text{-}C_{60}) = -0.42$ eV; the driving force for the charge recombination process of the s-ET product is $\Delta G_{CR(s\text{-}ET)} = E(C_{60}\text{-}IT\text{-}C_{60}) - E(C_{60}^{-\bullet}\text{-}IT^{+\bullet}\text{-}C_{60}) = -1.34$ eV; the driving force for the m-ET process is $\Delta G_{CS(m\text{-}ET)} = E(C_{60}^{-\bullet}\text{-}IT^{2+}\text{-}C_{60}^{-\bullet}) - E({}^1C_{60}^*\text{-}IT\text{-}{}^1C_{60}^*) = -0.59$ eV; the driving force for the charge recombination process of the m-ET product is $\Delta G_{CR(m\text{-}ET)} = E(C_{60}\text{-}IT\text{-}C_{60}) - E(C_{60}^{-\bullet}\text{-}IT^{2+}\text{-}C_{60}^{-\bullet}) = -2.93$ eV. This indicates that both s-ET and m-ET are thermodynamically feasible, with m-ET having a greater driving force. All of the driving forces and energy levels involved in the s-ET and m-ET processes have been summarized in **Table S2**.

Calculation absorption spectrum of IT2 at the dicationic-state.

We calculated the absorption spectrum of the dicationic-state of **IT2** to assist in identifying the dication absorption peaks in the transient absorption spectra. To achieve this, based on the optimized structure of **IT2**, we added two positive charges to form the dicationic-state and optimized the dicationic-state structure of **IT2** using DFT at the B3LYP-D3BJ/6-31G* level. Using this optimized structure, we further calculated the first 50 vertically excited states using time-dependent DFT (TD-DFT) at the same level. On the basis of the calculated excitation transition energies and oscillator strengths, using the Multiwfn program (*9*), the absorption spectrum of the dicationic-state of **IT2** and extracted the visible region part can be obtained, as shown in **Fig. 3C** in the main text.

Comparison of the nanosecond transient absorption spectra of $^3C_{60}$* and IT^{2+}.

The nanosecond transient absorption spectrum in **Fig. 4E** should be attributed to the absorption of the dication IT^{2+}, rather than the localized triplet state of C_{60} ($^3C_{60}$*). Firstly, the femtosecond transient absorption measurement indicates that the excited-state absorption (ESA) around 760 nm forms within 1 ps, which is much faster than the time required to form the fullerene triplet state via intersystem crossing (ISC). To confirm this, we measured the femtosecond transient absorption spectra of **IT2** in toluene, where the low-polarity toluene results in the final single-electron recombination to $^3C_{60}$* (absorption around 690 nm) (*8*). As shown in **Fig. S10(A)**, it is evident that $^3C_{60}$* only begins to form after 1 ns, reflecting the intersystem crossing time, which is much slower than the time required for generating the ESA around 760 nm. Additionally, **Fig. S10(B)** compares the nanosecond transient absorption spectra of $^3C_{60}$* and IT^{2+}, with the former showing an absorption peak around 690 nm and the latter around 760 nm, highlighting a significant difference. Combining this with the chemical oxidation absorption spectra and DFT calculations, the nanosecond transient absorption spectrum of **IT2** in PhCN in **Fig. 4E** should be attributed to the multi-electron transfer product IT^{2+}.

Discussion of multi-electron transfer kinetics.

Due to the time resolution limit of the transient absorption spectrometer, it is challenging to distinguish if the m-ET constitutes stepwise processes. For instance, the following processes may exist. The initial electron-transfer occurs from the initial-state, $^1C_{60}*$-IT-$^1C_{60}*$ (denoted as species A), to the intermediate-state $C_{60}^{-\bullet}$-$IT^{+\bullet}$-$^1C_{60}*$ (denoted as species B). Subsequently, the second electron-transfer occurs from $C_{60}^{-\bullet}$-$IT^{+\bullet}$-$^1C_{60}*$ to the final charge-accumulated state $C_{60}^{-\bullet}$-IT^{2+}-$C_{60}^{-\bullet}$ (denoted as species C). The rate constant of the first electron transfer is denoted as k_1, and the rate constant of the second electron transfer is denoted as k_2. Thus, the multi-electron transfer process can be simplified as:

$$A \overset{k_1}{\rightarrow} B \overset{k_2}{\rightarrow} C$$

Denote the initial concentration of species A as $[A_0]$, and the concentrations of species A, B, and C at time t are [A], [B], and [C], respectively. According to first-order reaction kinetics, it follows:

$$\begin{cases} -\frac{d[A]}{dt} = k_1[A] \\ \frac{d[B]}{dt} = k_1[A] - k_2[B] \\ \frac{d[C]}{dt} = k_2[B] \end{cases}$$

According to the stoichiometric relationship, $[A_0]$ = [A]+[B]+[C], solving the aforementioned differential equation gives:

$$\begin{cases} [A] = [A_0]e^{-k_1 t} \\ [B] = \frac{k_1}{k_2 - k_1}[A_0](e^{-k_1 t} - e^{-k_2 t}) \\ [C] = [A_0](1 - \frac{k_2}{k_2 - k_1}e^{-k_1 t} + \frac{k_1}{k_2 - k_1}e^{-k_2 t}) \end{cases}$$

Assuming the existence of the intermediate state $C_{60}^{-\bullet}$-$IT^{+\bullet}$-$^1C_{60}*$, we should observe its transient absorption spectrum within 0.5 ps. However, we did not observe the absorption of this intermediate state in fact. Therefore, even if it exists, its lifetime is very short, possibly beyond the detection limit of the instrument. On the basis of the aforementioned kinetic analysis, k_2 should be much greater than k_1, $k_2 >> k_1$, so intermediate-state B ($C_{60}^{-\bullet}$-$IT^{+\bullet}$-$^1C_{60}*$) cannot be accumulated but rapidly convert to the product $C_{60}^{-\bullet}$-IT^{2+}-$C_{60}^{-\bullet}$. Hence, the concentration of the product can be approximated as:

$$[C] = [A_0](1 - e^{-k_1 t})$$

It is equivalent to a first-order process from the initial-state $^1C_{60}*$-IT-$^1C_{60}*$ to the final charge-accumulated state $C_{60}^{-\bullet}$-IT^{2+}-$C_{60}^{-\bullet}$. Therefore, the fitted rate constant, $(0.5\ ps)^{-1}$, approximate the rate constant of the total multi-electron transfer processes.

Calculation of the hole-transfer reorganization energy.

The total energies were calculated at the B3LYP-D3BJ/6-31G(d) level of theory with Grimme's D3 dispersion correction using Gaussian16 package (*10, 11*).

The internal reorganization energy for single-hole transfer (s-ET) can be calculated as (*12*): λ1 = E(cation at optimized neutral geometry) - E(optimized cation) + E(neutral geometry at optimized cation) - E(optimized neutral geometry). For **IT2**, the calculated λ1 is about 0.54 eV.

Similarly, the internal reorganization energy for two-hole transfer (m-ET) can be calculated as: λ2 = E(dication at optimized neutral geometry) - E(optimized dication) + E(neutral geometry at optimized dication) - E(optimized neutral geometry). For **IT2**, the calculated λ2 is about 0.4 eV.

Discussion between electron transfer kinetics and driving force.

Figs. S11 and **S12** illustrate the dependence of charge separation and recombination kinetics on solvent polarity during s-ET and m-ET processes. For s-ET, both charge separation and recombination accelerate with increasing solvent polarity. Similarly, for the m-ET process, although the dependence of charge separation rate on polarity is not significant, the charge recombination rate still accelerates with increasing polarity. In general, higher polarity provides greater driving force for charge separation and a smaller driving force for charge recombination, indicating that charge recombination in both single and multi-electron transfer processes occurs in the Marcus inverted region (*13, 14*). Thus, a greater driving force for charge recombination can lead to slower recombination, extending the lifetime of the multi-electron transfer products.

Transient absorption of IT2 triad mixed with the photocatalytic substrates.

Fig. S13 exhibits the nanosecond transient absorption spectra (ns-TA) of the triad **IT2** mixed with the catalytic reaction substrates. The concentration of **IT2** was 6×10^{-5} M, and the concentration of the catalytic substrates (THIQ or DTE^{red}) was 6×10^{-2} M (1000-fold excess). The ns-TA were conducted in PhCN solution. Upon 532 nm wavelength excitation, it can be observed the decay rate of the dication IT^{2+} around 760 nm significantly accelerated in systems containing photocatalytic substrates (**Fig. 6B** in the main text). Specifically, under 532 nm excitation, the decay lifetime of the dication in the pure **IT2** solution was approximately 12 μs (single-exponential fit), whereas in the system containing THIQ, the dication decay lifetime was only 0.86 μs, and in the system containing DTE^{red}, the dication decay lifetime was only 0.48 μs. This indicates that in photocatalytic multi-electron oxidation reactions, the dication was effectively quenched by the catalytic substrates (*15, 16*). We did not observe an enhanced excited-state signal of the catalytic substrates. On one hand, neither THIQ nor DTE^{red} absorb at 532 nm (or 550 nm); on the other hand, under 550 nm excitation in the femtosecond transient absorption spectra, THIQ and DTE^{red} showed no distinct excited-state absorption features (**Fig. S14**).

Photocatalytic oxidation of the 1,2,3,4-tetrahydroisoquinoline (THIQ).

Photocatalytic oxidation reactions were carried out at room temperature in an optical reactor with a 550 nm LED as the light source. The reaction substrate was a 0.1 M THIQ solution with PhCN as the solvent. The photocatalyst was **IT2** or **IT1** with a concentration of 10^{-4} M, which was one-thousandth of the reaction substrate. The reaction was carried out in air without adding any extra electron sacrificial agents. Under stirring conditions, timing started at the beginning of irradiation, and samples were taken at 3 h, 6 h, and 9 h to monitor the reaction processes by ^{1}H NMR. Chromatographically pure dichloromethane was used as an internal standard. The ^{1}H NMR spectra of the pristine THIQ and photocatalytic product DHIQ are shown in **Figs. S15** and **S16**. The ^{1}H NMR spectra of the reaction system at different times are recorded in **Figs. S19** and **S20**. The "control" spectrum is the ^{1}H NMR spectrum of 0.1 M THIQ irradiated for 9 h under the same conditions in the air atmosphere without photocatalyst. No detectable DHIQ product was formed, indicating that it was the photocatalysts **IT2** / **IT1**, rather than the oxygen, facilitating the conversion of THIQ to DHIQ. The chemical structures of THIQ and DHIQ, along with the reaction equation are as follows:

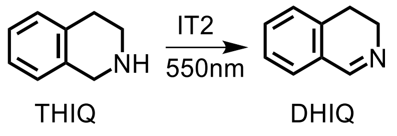

Photocatalytic oxidation of the dithioerythritol (DTE^{red}).

The experimental procedure for the photocatalytic oxidation of DTE^{red} is similar to that of the photocatalytic oxidation of THIQ. Briefly, the reaction substrate was a 0.1 M DTE^{red} solution with PhCN as the solvent. The photocatalyst was **IT2** or **IT1** at a concentration of 10^{-4} M, which is one-thousandth of the reaction substrate. Under stirring conditions, timing started at the beginning of irradiation, and samples were taken at 3 h, 6 h, and 9 h to monitor the reaction processes by ^{1}H-NMR. Chromatographically pure dichloromethane was used as an internal standard. The ^{1}H NMR spectra of the pristine DTE^{red} and photocatalytic product DTE^{ox} are shown in **Figs. S17** and **S18.** The ^{1}H NMR spectra of the reaction system at different times are recorded in **Figs. S21** and **S22**. The "control" spectrum is the ^{1}H NMR spectrum of 0.1 M DTE^{red} irradiated for 9 h under the same conditions in the air atmosphere without photocatalyst. No detectable DTE^{ox} product was formed, indicating that it was the photocatalysts **IT2** / **IT1**, rather than the oxygen, facilitating the conversion of DTE^{red} to DTE^{ox}. The chemical structures of DTE^{red} and DTE^{ox}, along with the reaction equation are as follows:

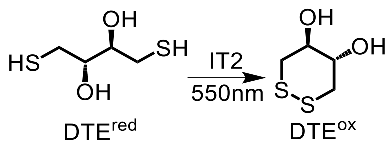

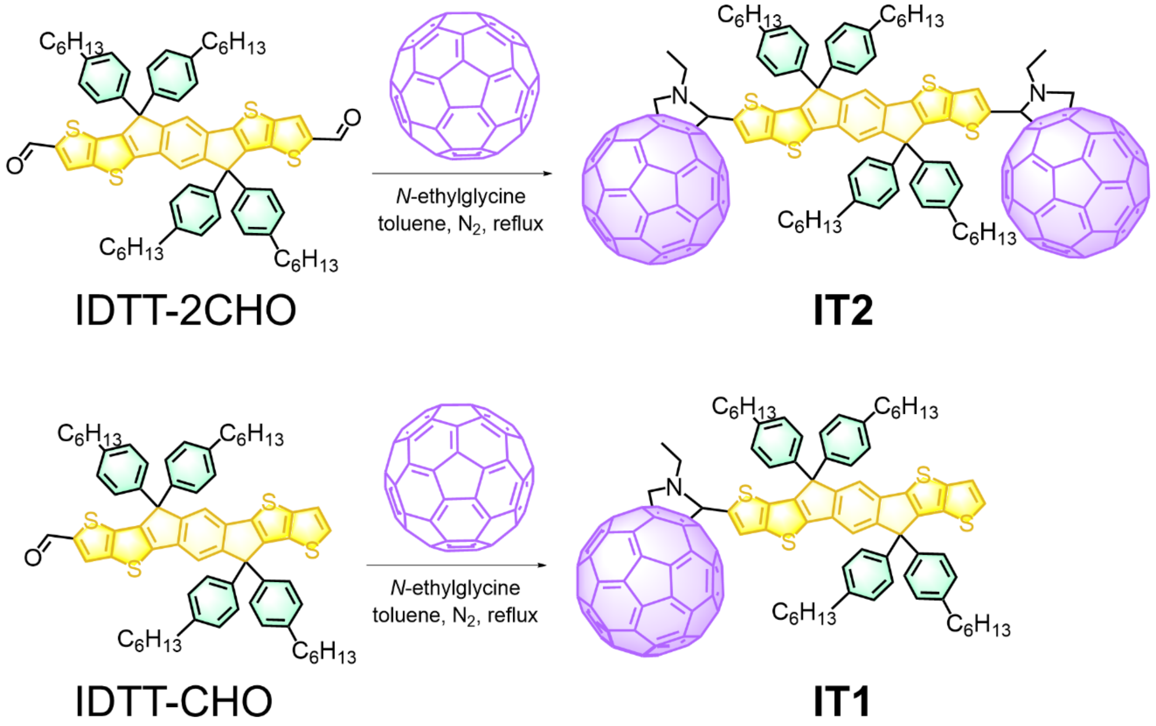

Fig. S1. Synthetic methodologies of **IT2** and **IT1**.

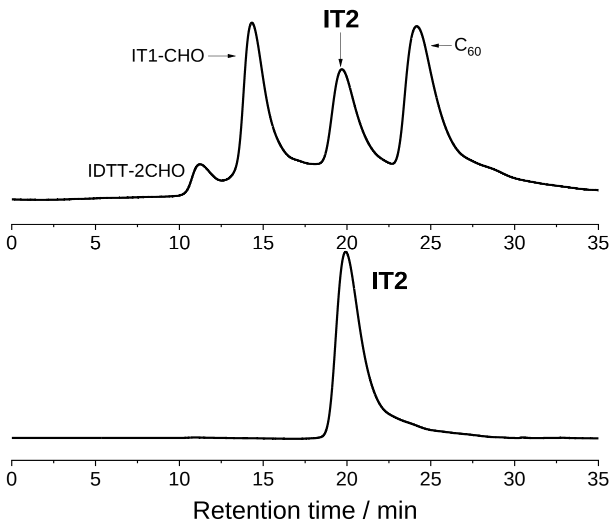

Fig. S2. HPLC profiles of raw reaction mixture (top) and purified **IT2** after separation (bottom). Buckprep column: 10 × 250 mm; eluent: toluene, 6 mL / min. Due to the introduction of the second C_{60}, **IT2** has a longer retention time on the buckprep column compared to **IT1** modified with a single fullerene.

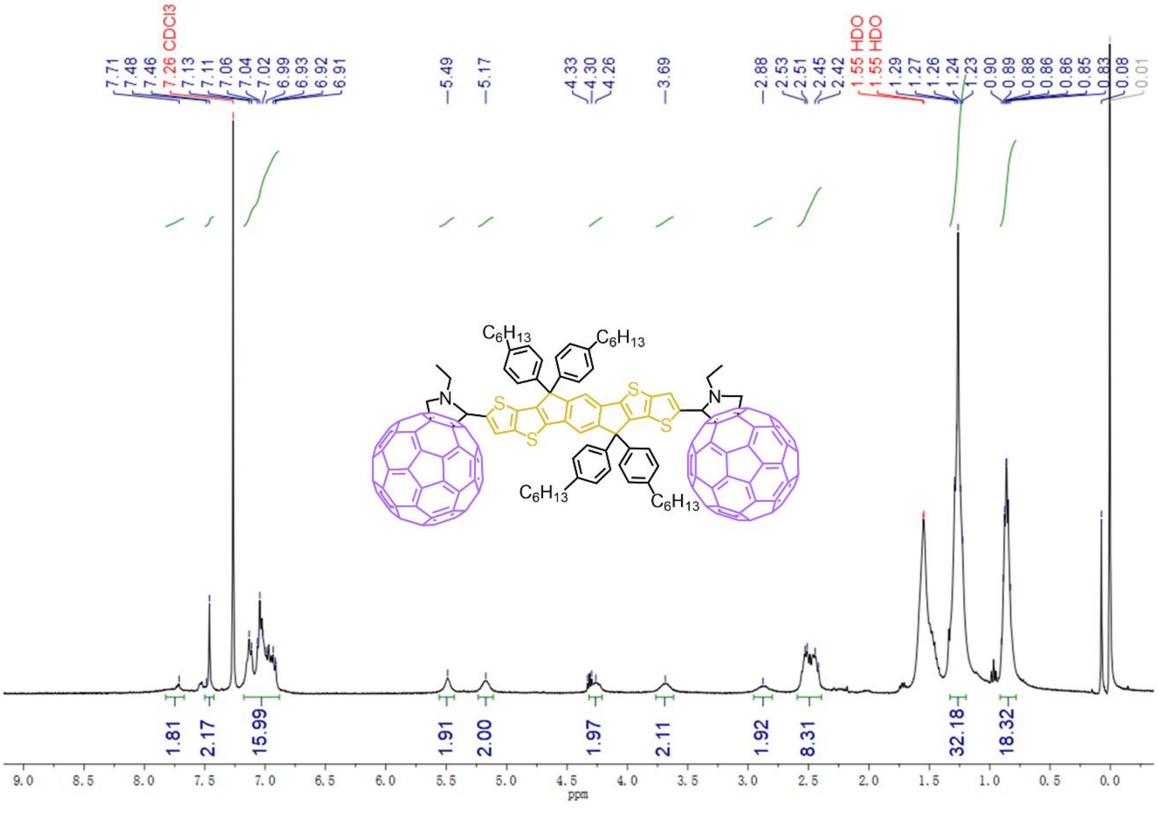

Fig. S3. ^{1}H NMR spectrum of **IT2** in $CDCl_3$.

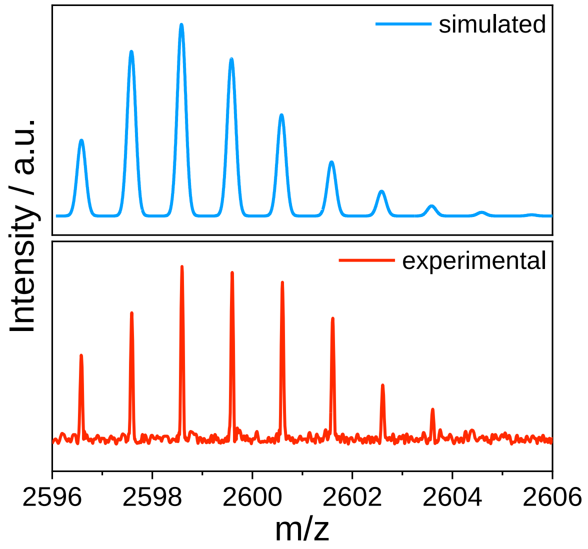

Fig. S4. Experimental high-mass-resolution MALDI-FT-ICR-MS spectrum and simulated spectrum of the isotope distribution of **IT2** molecular ion peak.

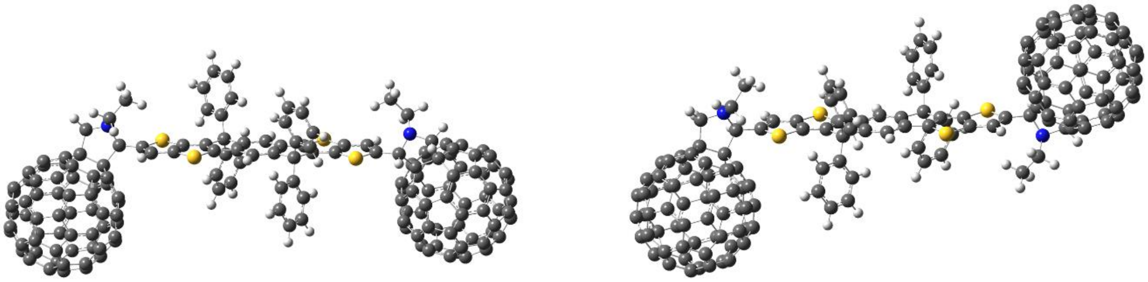

cis-configuration

0.00 eV

trans-configuration

+0.0085 eV

Fig. S5. Comparison of *cis*- and *trans*-configurations of **IT2** and their relative energies. Here the relative energy of the *cis*-configuration of **IT2** was set as 0 eV, and the calculated energy of the *trans*-configuration is about 8.5 meV higher than that of the *cis*-configuration.

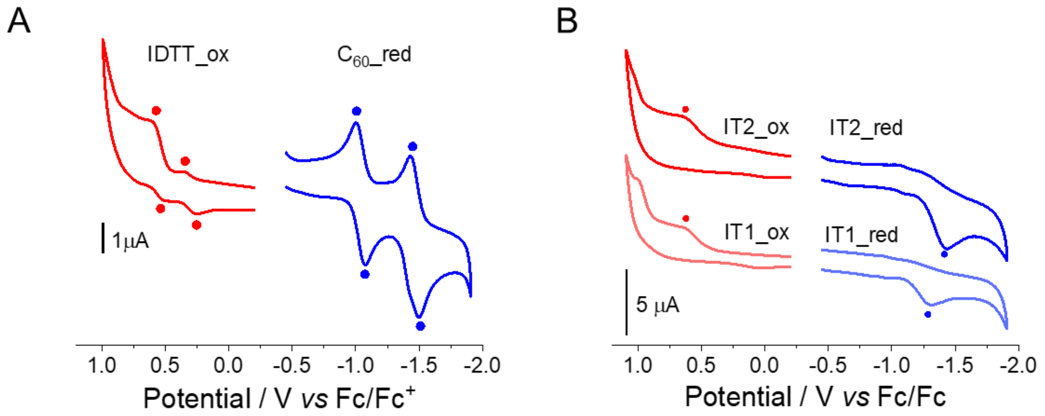

Fig. S6. Cyclic voltammetry curve (CV) of (A) the donor IDTT and acceptor C_{60}, and (B) **IT2** and **IT1** recorded in PhCN containing 0.05 M $TBAPF_6$. The ferrocene/ferrocenium (Fc/Fc^+) was used as the internal standard reference.

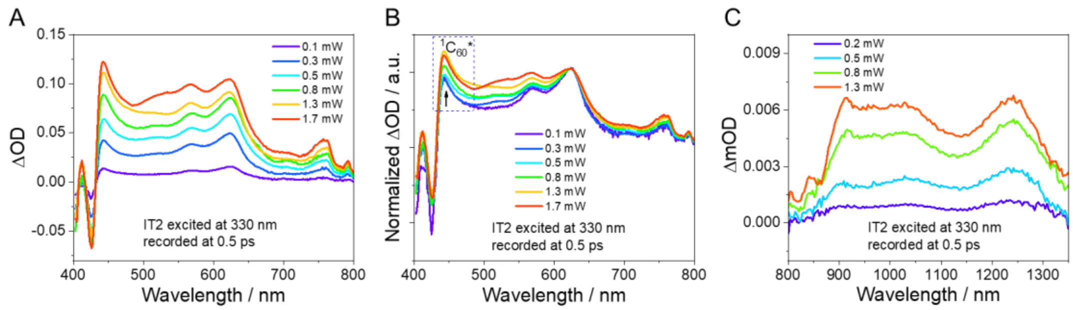

Fig. S7. (A) Transient absorption spectra (TAS) of **IT2** excited at 330 nm and recorded at 0.5 ps under various excitation powers in the visible region. (B) Normalized TAS of **IT2** excited at 330 nm and recorded at 0.5 ps under various excitation power. The excited-state absorption (ESA) around 450 nm is corresponding to the singlet state of C_{60} ($^1C_{60}$*). (C) Transient absorption spectra (TAS) of **IT2** excited at 330 nm and recorded at 0.5 ps under various excitation power in the NIR region. The ESA around 900 nm is corresponding to the singlet state of C_{60} ($^1C_{60}$*), and the normalized spectra in the NIR region has been exhibited in **Fig. 3H** in the main text, which more clearly demonstrates that the $^1C_{60}$* intensify with increasing excitation powers.

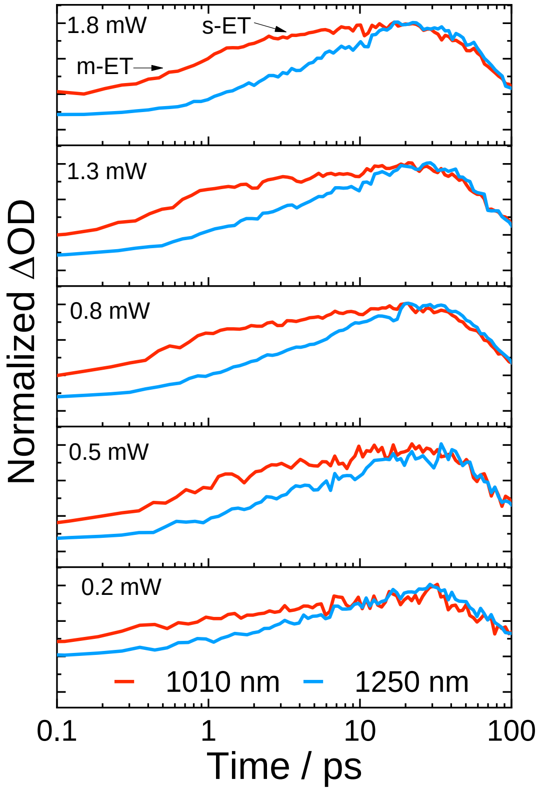

Fig. S8. Comparison of the C_{60} anion ($C_{60}^{-\bullet}$, recorded at 1010 nm) and IDTT cation ($IDTT^{+\bullet}$, recorded at 1250 nm) kinetics under 330 nm excitation at different excitation powers. In contrast to $IDTT^{+\bullet}$, the kinetics of $C_{60}^{-\bullet}$ display two formation processes: the slow process corresponds to the single-electron transfer; while the fast process corresponds to multi-electron transfer, with which features becoming more pronounced as the excitation power increases.

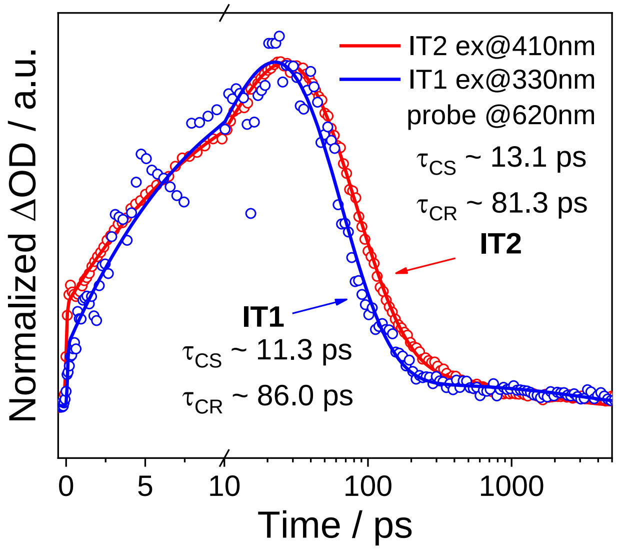

Fig. S9. Single-electron transfer kinetics for **IT2** under 410 nm excitation and for **IT1** under 330 nm excitation, probed at 620 nm for the IDTT cation absorption.

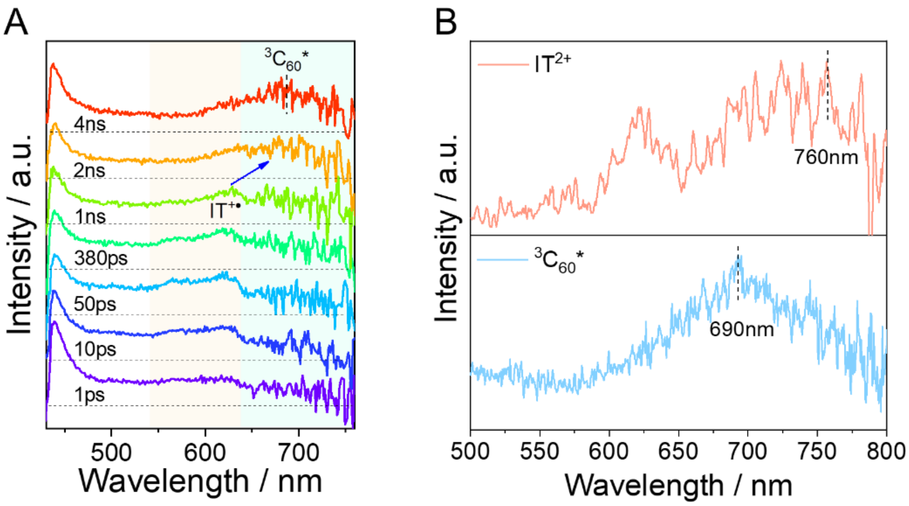

Fig. S10. (A) Spectral evolution of **IT2** in toluene from the charge-separated state ($IT^{+\bullet}$) to the C_{60} triplet excited state ($^3C_{60}$*) within 5 ns obtained from the femtosecond transient absorption. (B) Comparison of the absorption spectra of IT^{2+} (top) and $^3C_{60}$* (bottom) obtained from the nanosecond transient absorption.

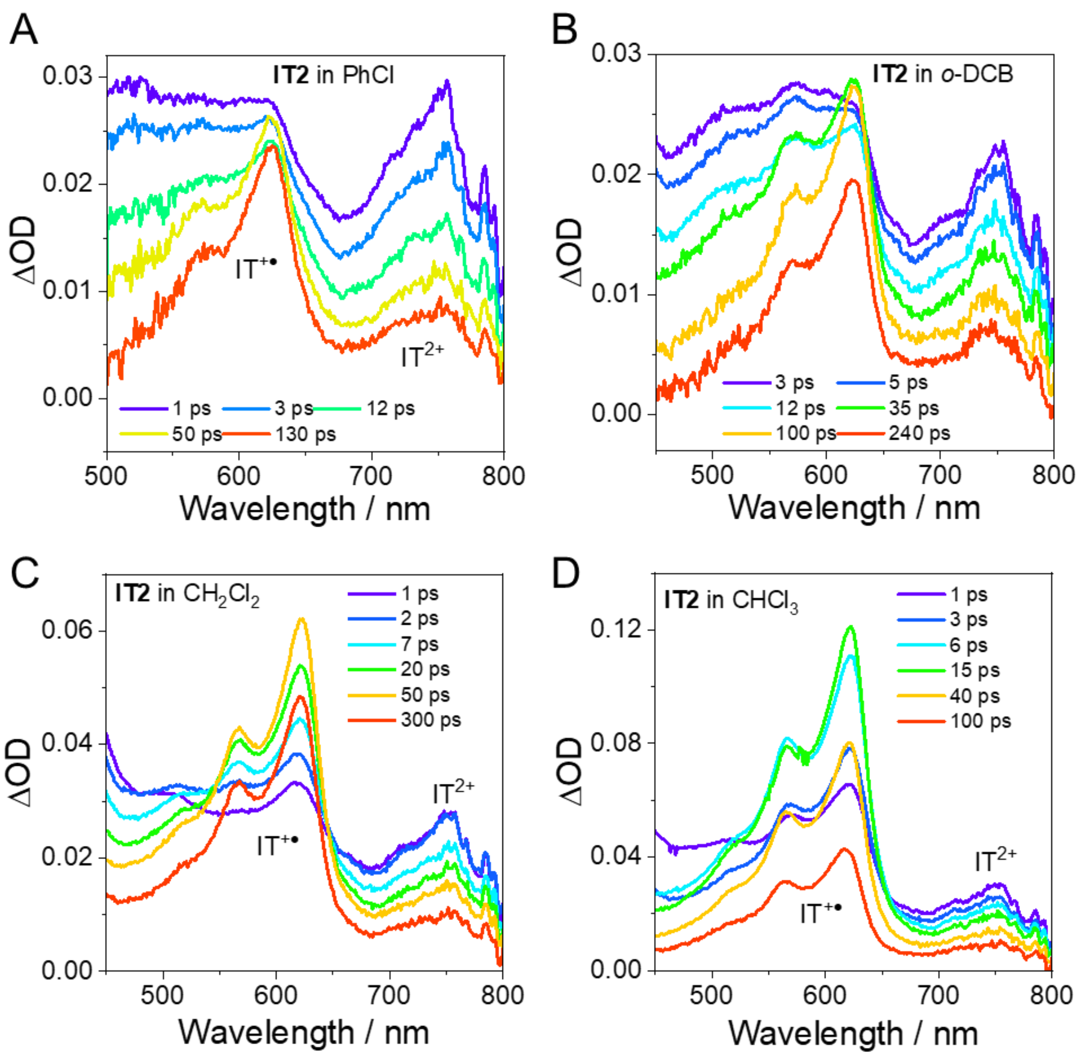

Fig. S11. Selected transient absorption spectra (TAS) following 330 nm excitation (~1 mW) for **IT2** in (A) chlorobenzene (PhCl), (B) *o*-dichlorobenzene (*o*-DCB), (C) dichloromethane (CH_2Cl_2), and (D) chloroform ($CHCl_3$). The excited-state absorption (ESA) around 625 nm can be attributed to the cation ($IT^{+•}$) absorption, and the ESA around 760 nm attributes to the dication (IT^{2+}) absorption, according to the chemical oxidation absorption.

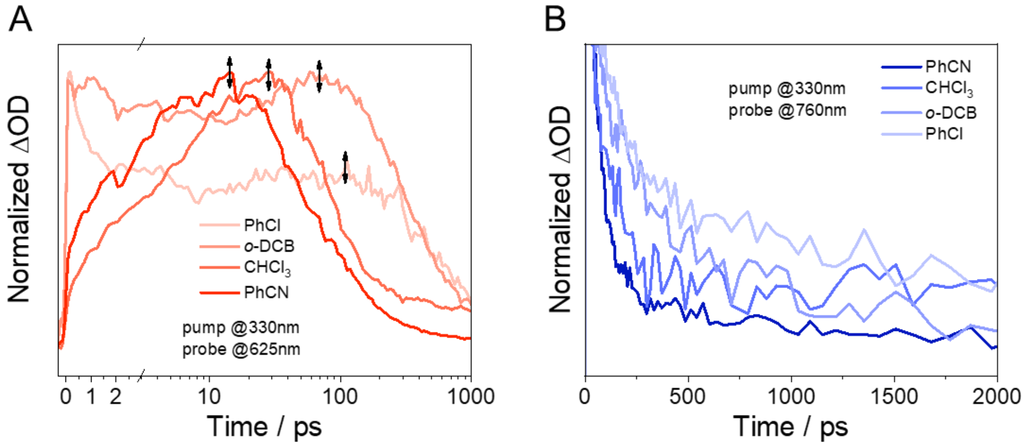

Fig. S12. Normalized electron transfer kinetics for **IT2** in chlorobenzene (PhCl), *o*-dichlorobenzene (*o*-DCB), chloroform ($CHCl_3$) and benzonitrile (PhCN), exciting at 330 nm and probing at (A) 625 nm for the cation absorption and (B) 760 nm for the dication absorption.

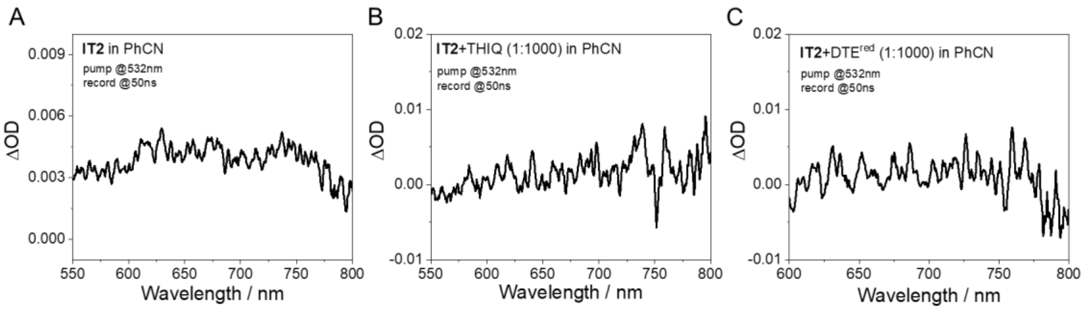

Fig. S13. Transient absorption (TA) analysis of **IT2** mixed with photocatalytic substrate. Selected TA spectrum at 50 ns after 532 nm excitation in PhCN for (A) **IT2**, (B) **IT2** mixed with 1000-fold excess THIQ, and (C) **IT2** mixed with 1000-fold excess DTE^{red}.

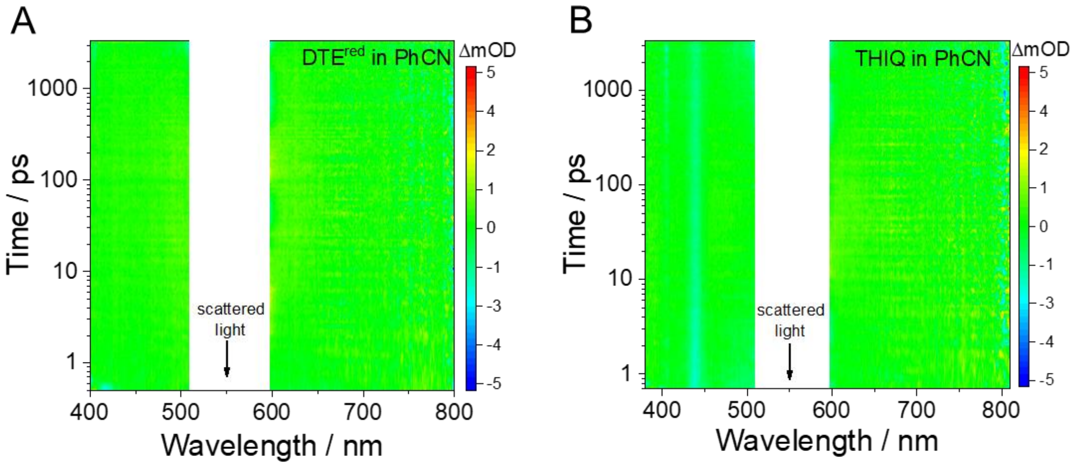

Fig. S14. Contour plot of the femtosecond transient absorption of (A) DTE^{red} and (B) THIQ in PhCN following 550 nm excitation.

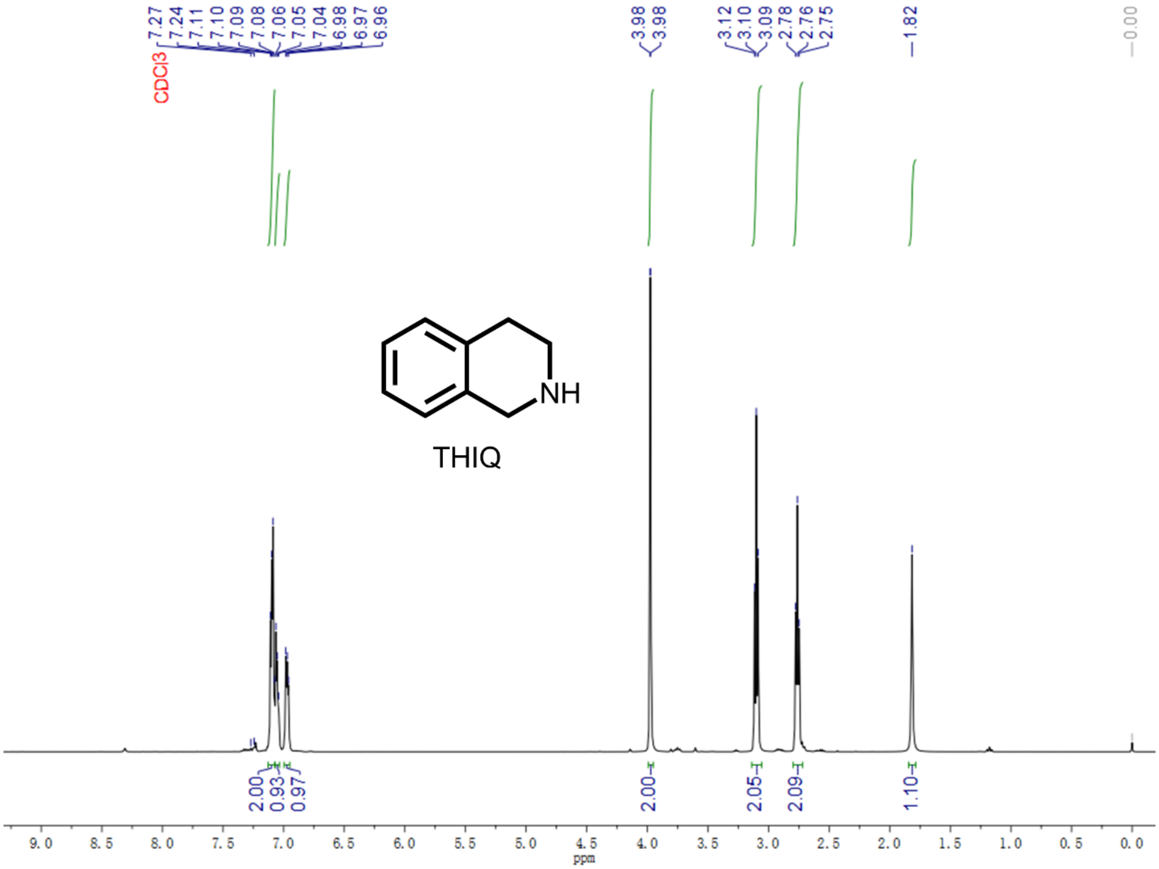

Fig. S15. ^{1}H NMR spectrum of photocatalytic substrate 1,2,3,4-tetrahydroisoquinoline (THIQ) measured in $CDCl_3$.

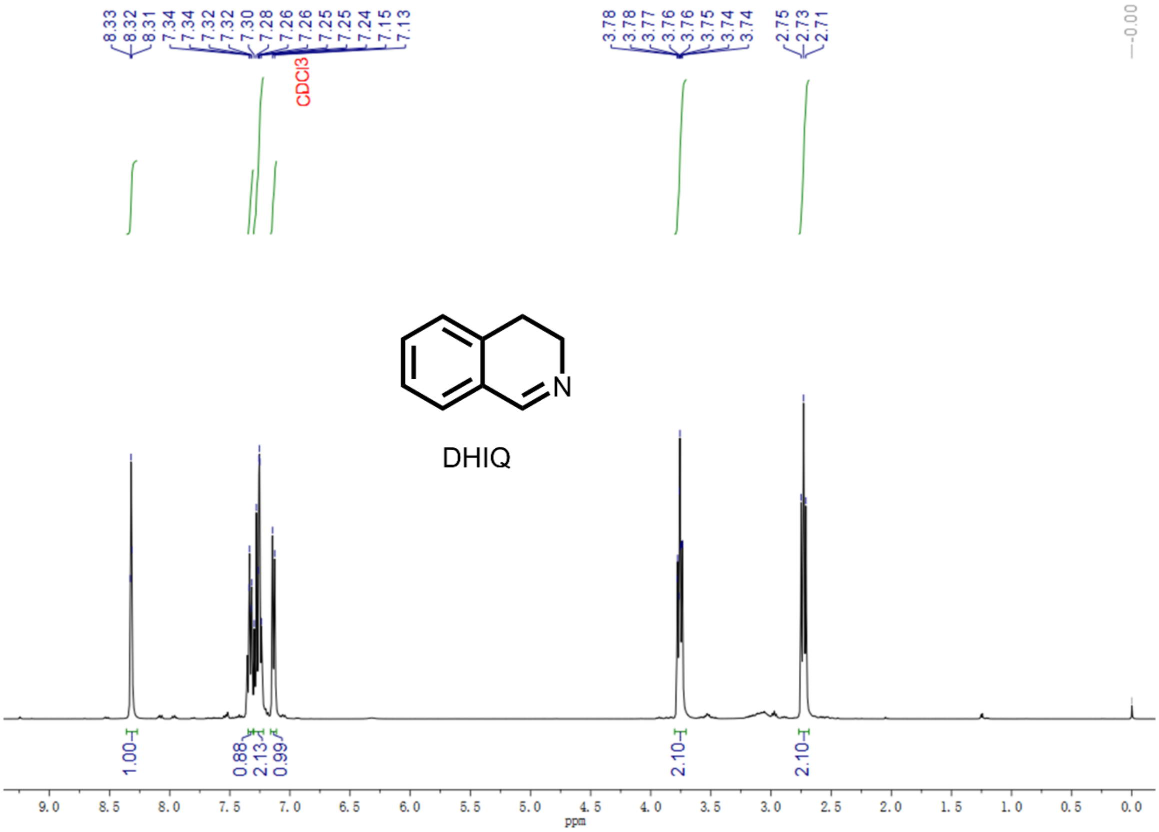

Fig. S16. ^1H NMR spectrum of photocatalytic product 3,4-dihydroisoquinoline (DHIQ) measured in $CDCl_3$.

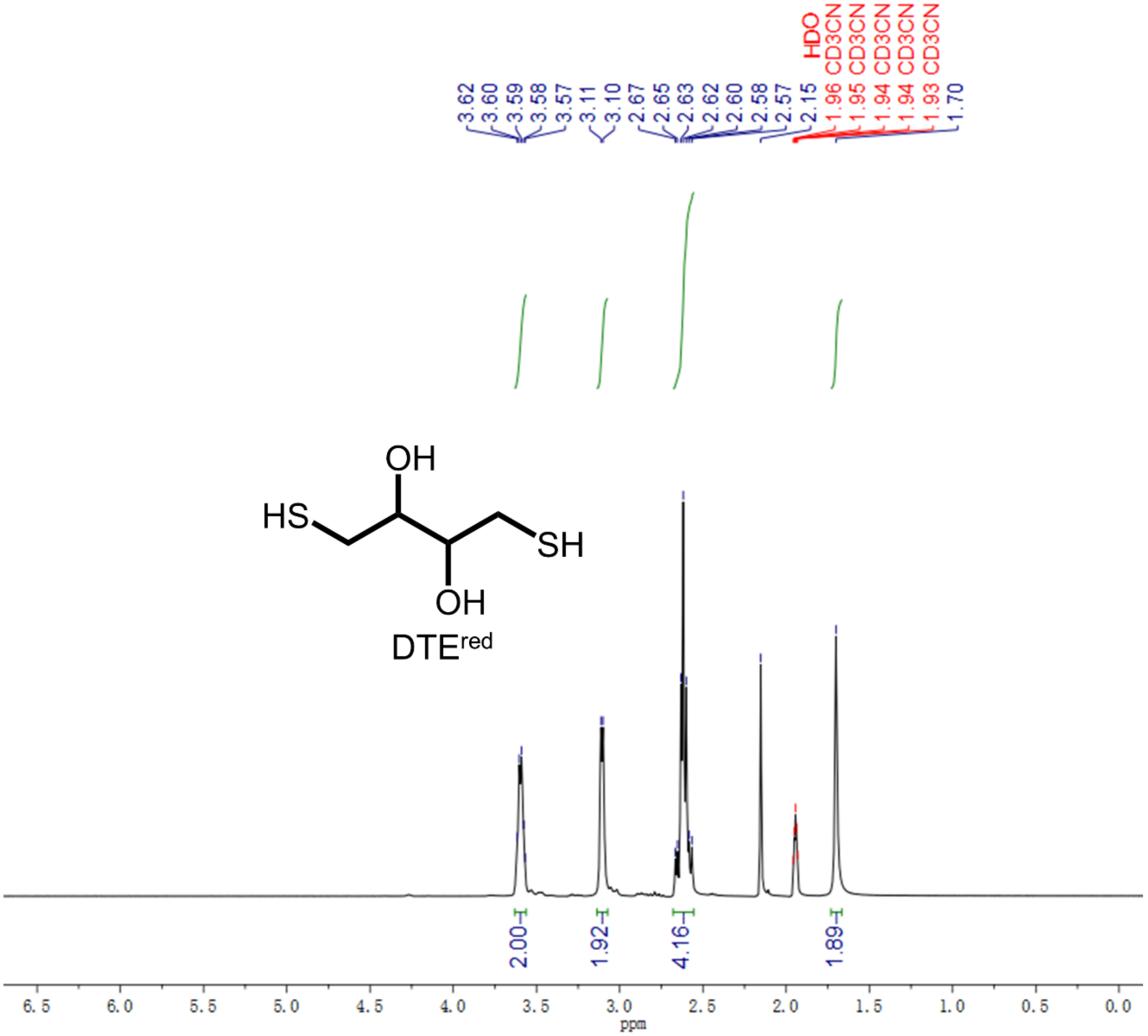

Fig. S17. ^{1}H NMR spectrum of photocatalytic substrate dithioerythritol (DTE^{red}) measured in CD_3CN.

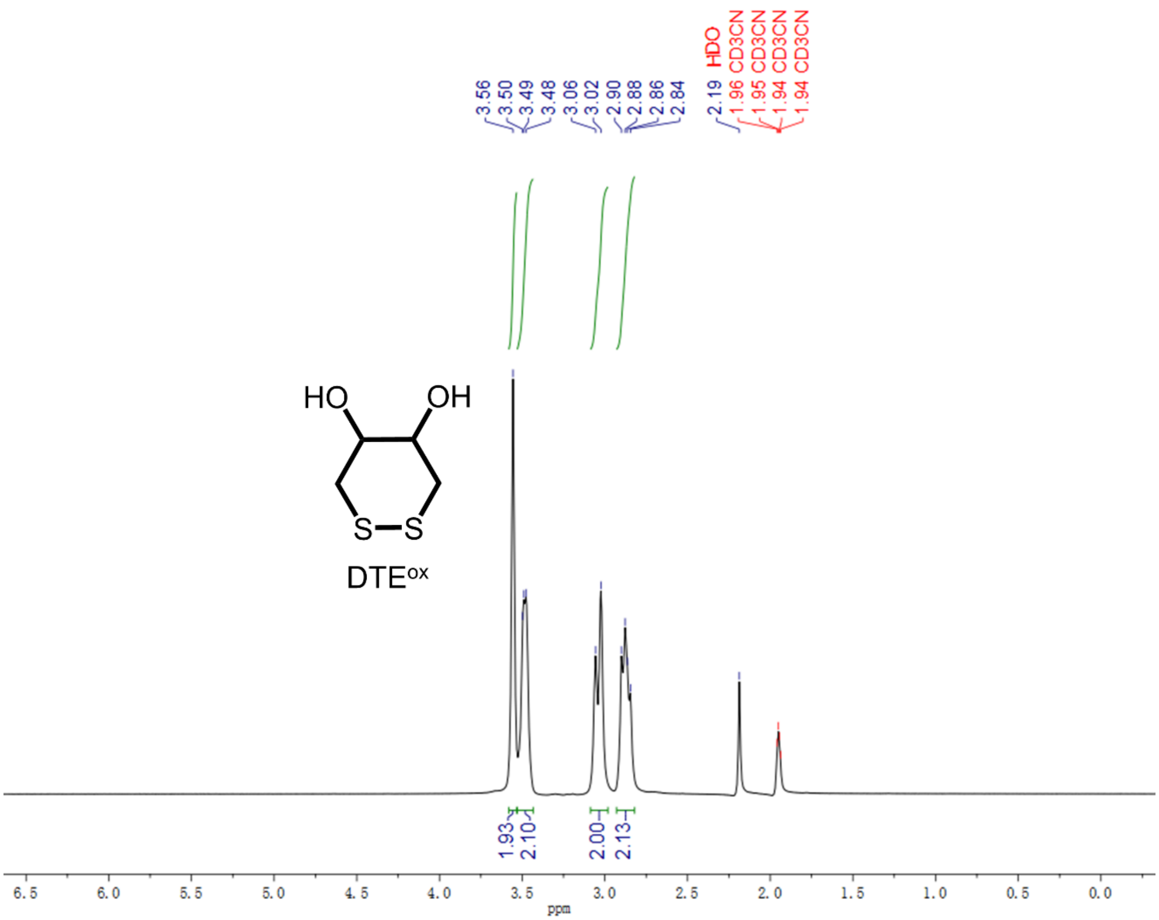

Fig. S18. ^{1}H NMR spectrum of photocatalytic product 4,5-dihydroxy-1,2-dithiane (DTE^{ox}) measured in CD_3CN.

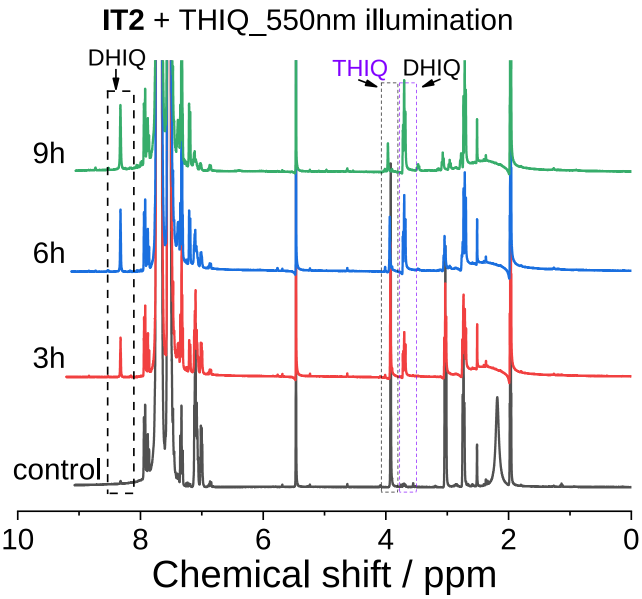

Fig. S19. Changes in the ^{1}H NMR spectrum during the photocatalytic oxidation of 1,2,3,4-tetrahydroisoquinoline (THIQ) by **IT2** under different 550 nm illumination times. The "control" spectrum is the ^{1}H NMR spectrum of THIQ irradiated with 550 nm light for 9 hours under the same conditions without the **IT2** photocatalyst. The experiment was conducted in benzonitrile (PhCN) and deuterated chloroform ($CDCl_3$), with chromatographically pure dichloromethane as the internal standard. The peaks around chemical shift of 7.5 ppm corresponds to the protons of the solvent PhCN, and the peak around a chemical shift of 5.5 ppm corresponds to the protons of the internal standard dichloromethane.

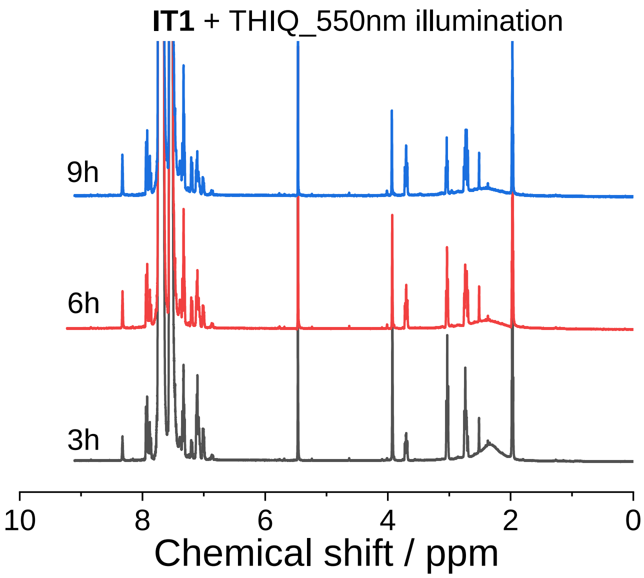

Fig. S20. Changes in the ^{1}H NMR spectrum during the photocatalytic oxidation of 1,2,3,4-tetrahydroisoquinoline (THIQ) by **IT1** under different 550 nm illumination times. The experiment was conducted in benzonitrile (PhCN) and deuterated chloroform ($CDCl_3$), with chromatographically pure dichloromethane as the internal standard. The peaks around chemical shift of 7.5 ppm corresponds to the protons of the solvent PhCN, and the peak around a chemical shift of 5.5 ppm corresponds to the protons of the internal standard dichloromethane.

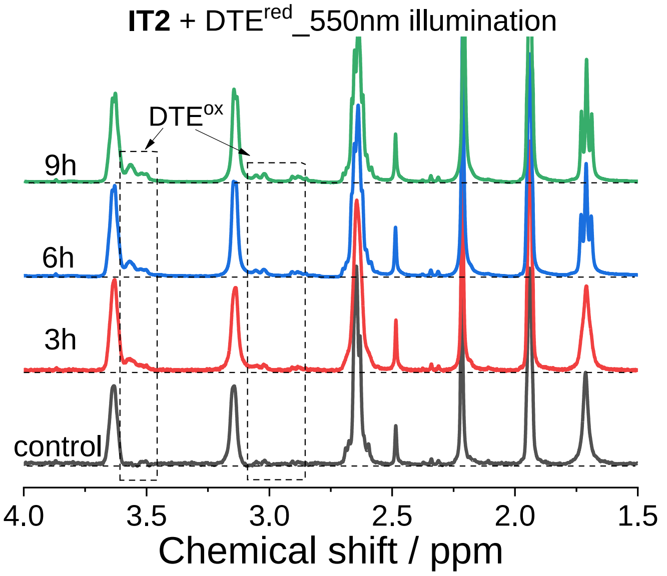

Fig. S21. Changes in the ^{1}H NMR spectrum during the photocatalytic oxidation of dithiothreitol (DTE^{red}) by **IT2** under different 550 nm illumination times. The "control" spectrum is the ^{1}H NMR spectrum of THIQ irradiated with 550 nm light for 9 hours under the same conditions without the **IT2** photocatalyst. The experiment was conducted in benzonitrile (PhCN) and deuterated acetonitrile (CD_3CN), with chromatographically pure dichloromethane as the internal standard.

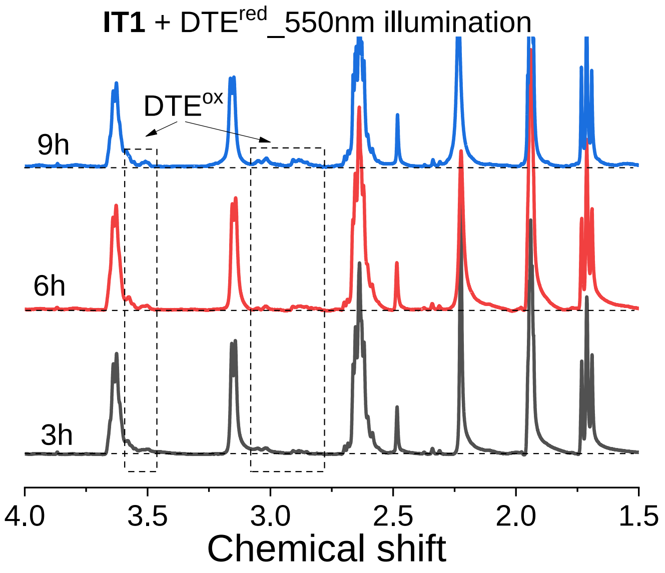

Fig. S22. Changes in the ^{1}H NMR spectrum during the photocatalytic oxidation of dithiothreitol (DTE^{red}) by **IT1** under different 550 nm illumination times. The experiment was conducted in benzonitrile (PhCN) and deuterated acetonitrile (CD_3CN), with chromatographically pure dichloromethane as the internal standard.

Table S1. Redox potentials of the IDTT donor and C_{60} acceptor obtained from cyclic voltammetry (CV) testing, with Fc/Fc^+ as the internal standard.

Molecules	$^{2}E_{ox}$ (V vs Fc/Fc^+)	$^{1}E_{ox}$ (V vs Fc/Fc^+)	$^{1}E_{red}$ (V vs Fc/Fc^+)
C_{60}	-	-	-1.039
IDTT	0.553	0.297	-

Table S2. Energy levels estimation of the excited states and single-/multi-electron transfer states based on electrochemical results.

Excited states	Energy level / eV	ΔG_{CS} / eV	ΔG_{CR} / eV
C_{60}-IT-C_{60}	0	–	–
$^1C_{60}$*-IT-C_{60}	1.76	–	–
$C_{60}^{-\bullet}$-$IT^{+\bullet}$-C_{60}	1.34	-0.42	-1.34
$^1C_{60}$*-IT-$^1C_{60}$*	3.52	–	–
$C_{60}^{-\bullet}$-IT^{2+}-$C_{60}^{-\bullet}$	2.93	-0.59	-2.93

Table S3. Summaries of the charge separation (CS) and charge recombination (CR) time constants (τ) during single-electron transfer (s-ET) or multi-electron transfer (m-ET) for **IT2** and **IT1**.

molecules	pump @330nm				pump @410nm	
	m-ET		s-ET		s-ET	
	τCS	τCR	τCS	τCR	τCS	τCR
**IT2**	0.5 ps	10.2 μs	11.4 ps	71.2 ps	13.1 ps	81.3 ps
**IT1**	—		11.3 ps	86.0 ps	14.8 ps	81.0 ps

Table S4. Atomic coordinates of the optimized neutral structure of **IT2** using DFT at the B3LYP-D3BJ/6-31G* level. The calculated Gibbs free energy is -8279.761336 hartree at the neutral state.

No.	Atom	X	Y	Z	No.	Atom	X	Y	Z
1	C	-7.1825	2.27963	-1.1174	110	C	11.564	-2.5288	3.06613
2	C	-6.6894	2.29616	0.16624	111	C	10.1954	-2.1974	2.70508
3	C	-5.2712	2.33541	0.18831	112	C	9.48782	-3.0257	1.82702
4	C	-4.6787	2.34362	-1.0813	113	C	8.78453	-3.2363	-0.4072
5	C	-2.8001	2.34604	0.2686	114	C	8.81088	-2.6214	-1.6577
6	C	-3.2657	2.3401	-1.0292	115	C	9.7421	-3.0804	-2.6761
7	H	-7.3114	2.2835	1.04982	116	C	10.6129	-4.1351	-2.3991
8	S	-5.8936	2.3125	-2.3203	117	C	11.9971	-4.0695	-2.8393
9	S	-4.0681	2.34365	1.47204	118	C	12.8267	-4.6705	-1.8065
10	C	-2.1111	2.36132	-2.0207	119	C	14.0815	-4.1335	-1.5177
11	C	-1.3511	2.35408	0.29843	120	C	14.5198	-4.0079	-0.1363
12	C	-0.9115	2.3607	-1.0512	121	C	14.2839	-2.4252	2.22226
13	C	0.43878	2.34943	-1.3606	122	C	15.1592	-1.9955	1.14086
14	C	-0.4386	2.3493	1.36063	123	C	15.2729	-2.7704	-0.0141
15	C	0.91161	2.36066	1.05123	124	C	15.2993	-2.1293	-1.3209
16	C	1.3512	2.35416	-0.2984	125	C	14.5626	-2.9717	-2.249
17	H	0.77829	2.3354	-2.3916	126	C	13.7658	-2.3904	-3.2366
18	H	-0.7782	2.33517	2.39158	127	C	12.455	-2.9514	-3.5381
19	C	7.18264	2.27982	1.11743	128	C	11.5506	-1.8525	-3.824
20	C	6.68952	2.29644	-0.1662	129	C	10.2206	-1.9139	-3.4044
21	C	5.27137	2.33564	-0.1883	130	C	8.66606	-2.4292	0.79904
22	C	4.67879	2.34373	1.08134	131	C	8.72094	-1.1715	-1.7596
23	C	2.80023	2.34618	-0.2686	132	C	12.3711	-4.9807	0.59963
24	C	3.26583	2.34016	1.02919	133	C	10.6378	3.17689	2.01214
25	H	7.31159	2.28385	-1.0498	134	H	10.6306	3.02145	3.09764
26	S	5.8937	2.31256	2.32028	135	H	11.3747	3.94838	1.78275
27	S	4.06823	2.34393	-1.472	136	C	8.58844	2.28353	1.62809
28	C	2.11126	2.36125	2.02075	137	N	9.31058	3.55409	1.57862
29	C	-2.2266	3.65016	-2.8498	138	C	9.22563	4.33537	0.33182
30	C	-1.5788	4.82896	-2.4679	139	H	10.1982	4.81437	0.17951
31	C	-3.0825	3.68205	-3.9592	140	H	9.06161	3.69616	-0.5452
32	C	-1.7757	6.01111	-3.1826	141	C	8.14114	5.40766	0.41775
33	H	-0.917	4.8241	-1.6088	142	H	7.1547	4.96179	0.56759
34	C	-3.2824	4.86306	-4.6712	143	H	8.34181	6.07881	1.2587
35	H	-3.5829	2.77256	-4.276	144	H	8.11381	5.9985	-0.5055
36	C	-2.6276	6.03376	-4.2864	145	C	-11.505	1.8266	1.1908
37	H	-1.2593	6.91563	-2.8734	146	C	-10.176	1.252	1.49323
38	H	-3.9499	4.866	-5.5282	147	C	-9.3421	0.85045	0.47316
39	H	-2.7792	6.95419	-4.8429	148	C	-9.5646	1.17629	-1.0049
40	C	-2.0458	1.10316	-2.8958	149	C	-11.002	1.79217	-1.3305
41	C	-2.703	-0.0726	-2.521	150	C	-11.918	1.95495	-0.1159
42	C	-1.2597	1.09553	-4.0557	151	C	-12.425	1.35886	2.20811
43	C	-2.5807	-1.2308	-3.2898	152	C	-11.691	0.51264	3.13426
44	H	-3.3128	-0.0841	-1.624	153	C	-10.307	0.44983	2.6928
45	C	-1.1338	-0.0615	-4.8217	154	C	-9.5844	-0.7386	2.83453
46	H	-0.759	2.00628	-4.3692	155	C	-8.6289	-0.3953	0.60088
47	C	-1.7956	-1.2306	-4.4419	156	C	-9.2967	-0.1894	-1.6294
48	H	-3.1037	-2.133	-2.9853	157	C	-10.088	-0.7561	-2.604
49	H	-0.5216	-0.0476	-5.7191	158	C	-11.42	-0.1856	-2.909

50	H	-1.7025	-2.1314	-5.0417	159	C	-11.878	0.91708	-2.2221
51	C	2.22667	3.65002	2.84994	160	C	-13.242	0.95238	-1.7682
52	C	1.57879	4.82883	2.4681	161	C	-13.268	1.59346	-0.4653
53	C	3.08256	3.68184	3.9593	162	C	-14.18	1.16594	0.50538
54	C	1.77571	6.01092	3.1829	163	C	-13.747	1.04456	1.87874
55	H	0.91704	4.82402	1.60907	164	C	-12.299	-0.6107	3.69291
56	C	3.2824	4.86279	4.67145	165	C	-13.671	-0.9451	3.33663
57	H	3.58304	2.77234	4.2761	166	C	-14.379	-0.1339	2.44851
58	C	2.62754	6.0335	4.28672	167	C	-15.211	-0.7397	1.41847
59	H	1.25922	6.91545	2.87377	168	C	-15.089	0.06604	0.21395
60	H	3.9499	4.86568	5.52851	169	C	-15.064	-0.548	-1.037
61	H	2.77909	6.95389	4.84333	170	C	-14.129	-0.0908	-2.0547
62	C	2.04604	1.10301	2.89575	171	C	-13.652	-1.2431	-2.7841
63	C	2.70319	-0.0727	2.52082	172	C	-12.315	-1.2901	-3.1916
64	C	1.2599	1.09524	4.05566	173	C	-8.5979	-1.0357	-0.7011
65	C	2.58095	-1.231	3.2895	174	C	-11.44	-4.5197	-1.6137
66	H	3.31306	-0.0841	1.62382	175	C	-10.126	-4.205	-1.2659
67	C	1.13408	-0.0619	4.82149	176	C	-9.6902	-4.3366	0.11734
68	H	0.7592	2.00595	4.36921	177	C	-10.587	-4.7764	1.09245
69	C	1.79594	-1.2309	4.44158	178	C	-11.955	-5.1057	0.72659
70	H	3.104	-2.1331	2.98489	179	C	-13.682	-4.4197	-0.9012
71	H	0.52186	-0.0481	5.71894	180	C	-13.56	-3.6123	-2.1015
72	H	1.70288	-2.1318	5.04133	181	C	-12.173	-3.6673	-2.5393
73	C	11.505	1.82658	-1.1908	182	C	-11.564	-2.5288	-3.0661
74	C	10.1756	1.25205	-1.4932	183	C	-10.196	-2.1974	-2.7051
75	C	9.34212	0.85055	-0.4731	184	C	-9.488	-3.0258	-1.827
76	C	9.56463	1.17639	1.00487	185	C	-8.7847	-3.2364	0.4072
77	C	11.0018	1.79218	1.33048	186	C	-8.811	-2.6215	1.65771
78	C	11.9177	1.95491	0.11586	187	C	-9.7423	-3.0805	2.67615
79	C	12.425	1.35879	-2.2081	188	C	-10.613	-4.1352	2.39907
80	C	11.6906	0.5126	-3.1343	189	C	-11.997	-4.0694	2.83926
81	C	10.3074	0.44987	-2.6928	190	C	-12.827	-4.6704	1.80644
82	C	9.58435	-0.7385	-2.8345	191	C	-14.082	-4.1334	1.51767
83	C	8.62885	-0.3951	-0.6009	192	C	-14.52	-4.0077	0.13623
84	C	9.29668	-0.1893	1.62941	193	C	-14.284	-2.425	-2.2223
85	C	10.0881	-0.756	2.604	194	C	-15.159	-1.9953	-1.1409
86	C	11.42	-0.1856	2.909	195	C	-15.273	-2.7702	0.0141
87	C	11.8783	0.91705	2.22209	196	C	-15.299	-2.129	1.32083
88	C	13.2425	0.95227	1.76817	197	C	-14.563	-2.9715	2.24897
89	C	13.2676	1.59335	0.46529	198	C	-13.766	-2.3903	3.2366
90	C	14.1798	1.16577	-0.5054	199	C	-12.455	-2.9513	3.53813
91	C	13.7473	1.04442	-1.8788	200	C	-11.551	-1.8524	3.82401
92	C	12.2993	-0.6108	-3.6929	201	C	-10.221	-1.914	3.40445
93	C	13.6707	-0.9453	-3.3367	202	C	-8.6662	-2.4294	-0.799
94	C	14.3789	-0.1341	-2.4486	203	C	-8.721	-1.1716	1.75967
95	C	15.2107	-0.7399	-1.4185	204	C	-12.371	-4.9806	-0.5996
96	C	15.0886	0.06583	-0.214	205	C	-10.638	3.17685	-2.0121
97	C	15.0641	-0.5482	1.03696	206	H	-10.63	3.02141	-3.0977
98	C	14.1289	-0.091	2.05466	207	H	-11.374	3.94838	-1.7828
99	C	13.6519	-1.2432	2.7841	208	C	-8.5883	2.28338	-1.6281
100	C	12.3145	-1.2902	3.19157	209	H	-8.5466	2.01527	-2.6917

101	C	8.59783	-1.0355	0.70111	210	N	-9.3104	3.55399	-1.5786
102	C	11.4403	-4.5197	1.61372	211	C	-9.2254	4.33526	-0.3318
103	C	10.1257	-4.205	1.26588	212	H	-10.198	4.81431	-0.1795
104	C	9.68998	-4.3365	-0.1173	213	H	-9.0614	3.69604	0.54521
105	C	10.5865	-4.7763	-1.0924	214	C	-8.1408	5.4075	-0.4177
106	C	11.9543	-5.1058	-0.7266	215	H	-7.1544	4.96158	-0.5676
107	C	13.6818	-4.4198	0.90119	216	H	-8.3415	6.07865	-1.2587
108	C	13.5602	-3.6124	2.1015	217	H	-8.1135	5.99834	0.50548
109	C	12.1727	-3.6674	2.53931	218	H	8.54677	2.01543	2.69171

Table S5. Atomic coordinates of the optimized cationic structure of **IT2** using DFT at the B3LYP-D3BJ/6-31G* level. The calculated Gibbs free energy is -8279.550056 hartree at the cationic state.

No.	Atom	X	Y	Z	No.	Atom	X	Y	Z
1	C	-7.2034	3.59094	0.24875	110	C	9.64596	-1.5819	3.34208
2	C	-6.4749	3.63466	1.42744	111	C	8.51923	-0.7053	3.06967
3	C	-5.0994	3.79327	1.18349	112	C	7.24767	-1.2507	2.86043
4	C	-4.7614	3.84943	-0.1886	113	C	5.69444	-1.8245	1.18964
5	C	-2.6676	3.90435	0.79916	114	C	5.46808	-1.8289	-0.1867
6	C	-3.386	3.89116	-0.4058	115	C	5.63513	-3.0564	-0.9481
7	H	-6.9071	3.50884	2.40825	116	C	6.02506	-4.2289	-0.2988
8	S	-6.1866	3.75956	-1.1752	117	C	7.00981	-5.1065	-0.9103
9	S	-3.6802	3.84617	2.22342	118	C	7.85104	-5.6453	0.14697
10	C	-2.4502	3.88303	-1.5998	119	C	9.21543	-5.833	-0.074
11	C	-1.2689	3.92088	0.55029	120	C	10.1766	-5.4843	0.96094
12	C	-1.0909	3.92399	-0.8772	121	C	11.5779	-3.3171	2.16885
13	C	0.16582	3.91599	-1.4307	122	C	12.0416	-3.8701	0.90435
14	C	-0.1658	3.91592	1.43062	123	C	11.354	-4.9302	0.31298
15	C	1.09098	3.92401	0.8771	124	C	11.1204	-4.9348	-1.1239
16	C	1.26892	3.92095	-0.5504	125	C	9.79895	-5.4923	-1.362
17	H	0.3082	3.89617	-2.5061	126	C	8.99412	-4.9715	-2.3761
18	H	-0.3081	3.89604	2.50602	127	C	7.56905	-4.7737	-2.1449
19	C	7.20341	3.59096	-0.2487	128	C	7.16955	-3.5512	-2.8177
20	C	6.47496	3.6347	-1.4274	129	C	6.22	-2.7094	-2.2358
21	C	5.09951	3.79337	-1.1835	130	C	6.4126	-0.7182	1.80836
22	C	4.76145	3.84952	0.18854	131	C	5.95298	-0.7273	-1.0081
23	C	2.66762	3.90446	-0.7992	132	C	8.30837	-4.7574	2.40407
24	C	3.38609	3.89125	0.40573	133	C	10.8162	2.83199	-0.3313
25	H	6.90717	3.50887	-2.4082	134	H	11.119	3.12471	0.68103
26	S	6.18665	3.75958	1.1752	135	H	11.6818	2.93218	-0.9873
27	S	3.68032	3.84629	-2.2235	136	C	8.61089	3.16429	0.02222
28	C	2.45027	3.88307	1.59972	137	N	9.71947	3.68147	-0.7617
29	C	-2.7493	5.13031	-2.4441	138	C	9.57097	3.84359	-2.219
30	C	-2.09	6.34131	-2.2128	139	H	10.5461	3.63043	-2.6659
31	C	-3.7844	5.09093	-3.388	140	H	8.88504	3.10457	-2.6534
32	C	-2.4472	7.48856	-2.922	141	C	9.16274	5.26978	-2.5846
33	H	-1.2942	6.39338	-1.4773	142	H	8.21343	5.55311	-2.1214
34	C	-4.1443	6.2382	-4.0924	143	H	9.92399	5.9745	-2.2375
35	H	-4.2959	4.15466	-3.5876	144	H	9.05982	5.37288	-3.6709
36	C	-3.4749	7.44139	-3.8633	145	C	-9.7049	0.18208	2.48887
37	H	-1.9205	8.41983	-2.7357	146	C	-8.2568	0.37719	2.25088
38	H	-4.9455	6.18925	-4.8236	147	C	-7.8132	0.90264	1.05988
39	H	-3.752	8.3343	-4.4153	148	C	-8.7243	1.54953	0.01554
40	C	-2.5292	2.57087	-2.3979	149	C	-10.285	1.34597	0.26655
41	C	-3.0333	1.40092	-1.8203	150	C	-10.619	0.52404	1.51674
42	C	-2.0034	2.51561	-3.6954	151	C	-9.8578	-1.0453	3.24306
43	C	-3.0161	0.19831	-2.5279	152	C	-8.5373	-1.607	3.47936
44	H	-3.4437	1.41817	-0.8159	153	C	-7.5537	-0.7291	2.86915
45	C	-1.9883	1.31467	-4.4019	154	C	-6.4125	-1.2696	2.26718
46	H	-1.6245	3.41955	-4.1618	155	C	-6.6613	0.32361	0.41673
47	C	-2.495	0.15132	-3.8198	156	C	-8.1871	0.91269	-1.2598
48	H	-3.4155	-0.6977	-2.0651	157	C	-8.9893	0.39722	-2.254

49	H	-1.5845	1.29012	-5.4098	158	C	-10.438	0.19884	-2.0222
50	H	-2.487	-0.7839	-4.3714	159	C	-10.996	0.52314	-0.8053
51	C	2.74925	5.13036	2.44404	160	C	-11.945	-0.3694	-0.1984
52	C	2.08992	6.34134	2.21279	161	C	-11.711	-0.3714	1.23527
53	C	3.78441	5.09102	3.38793	162	C	-11.892	-1.5491	1.96835
54	C	2.44709	7.48859	2.92202	163	C	-10.94	-1.8958	2.99893
55	H	1.29407	6.39338	1.47723	164	C	-8.3485	-2.9883	3.45984
56	C	4.1442	6.23829	4.09235	165	C	-9.4744	-3.8713	3.19197
57	H	4.29592	4.15477	3.58753	166	C	-10.743	-3.3351	2.96632
58	C	3.4748	7.44146	3.86332	167	C	-11.584	-3.8792	1.90996
59	H	1.92036	8.41984	2.73569	168	C	-12.297	-2.773	1.29145
60	H	4.94543	6.18938	4.8236	169	C	-12.521	-2.7692	-0.0838
61	H	3.75184	8.33438	4.41531	170	C	-12.35	-1.5409	-0.8457
62	C	2.5293	2.5709	2.39786	171	C	-11.774	-1.8775	-2.1271
63	C	3.03306	1.40089	1.82005	172	C	-10.824	-1.0239	-2.6966
64	C	2.00376	2.51566	3.69543	173	C	-6.8945	0.32734	-1.0167
65	C	3.01588	0.19826	2.52756	174	C	-8.1417	-3.5295	-3.1601
66	H	3.44323	1.41813	0.81551	175	C	-7.0564	-2.6906	-2.9025
67	C	1.98866	1.31469	4.40184	176	C	-6.0942	-3.0467	-1.8691
68	H	1.62503	3.41963	4.16192	177	C	-6.2589	-4.2242	-1.1383
69	C	2.49506	0.15129	3.81958	178	C	-7.3875	-5.0985	-1.4127
70	H	3.41498	-0.6978	2.06466	179	C	-9.7339	-4.955	-2.1738
71	H	1.58512	1.29016	5.40983	180	C	-10.447	-3.8495	-2.7877
72	H	2.48705	-0.7839	4.37118	181	C	-9.4628	-2.963	-3.392
73	C	9.70512	0.18209	-2.4887	182	C	-9.6464	-1.5818	-3.342
74	C	8.25699	0.37721	-2.2509	183	C	-8.5196	-0.7052	-3.0697
75	C	7.8133	0.90264	-1.0599	184	C	-7.248	-1.2506	-2.8606
76	C	8.72431	1.5495	-0.0155	185	C	-5.6946	-1.8245	-1.19
77	C	10.2849	1.34593	-0.2663	186	C	-5.4681	-1.8289	0.18631
78	C	10.6187	0.52402	-1.5165	187	C	-5.6351	-3.0565	0.94771
79	C	9.85808	-1.0453	-3.2429	188	C	-6.0251	-4.2289	0.29845
80	C	8.53769	-1.607	-3.4794	189	C	-7.0098	-5.1065	0.91006
81	C	7.55401	-0.7291	-2.8693	190	C	-7.8511	-5.6452	-0.1472
82	C	6.4127	-1.2695	-2.2674	191	C	-9.2155	-5.8329	0.07392
83	C	6.66129	0.32361	-0.4169	192	C	-10.177	-5.4843	-0.9609
84	C	8.18691	0.91264	1.25975	193	C	-11.578	-3.317	-2.1686
85	C	8.989	0.39714	2.25401	194	C	-12.042	-3.87	-0.904
86	C	10.4377	0.19874	2.02238	195	C	-11.354	-4.9301	-0.3128
87	C	10.9958	0.52306	0.80558	196	C	-11.12	-4.9348	1.12406
88	C	11.9449	-0.3695	0.19873	197	C	-9.7989	-5.4922	1.36199
89	C	11.7113	-0.3714	-1.2349	198	C	-8.9939	-4.9715	2.37609
90	C	11.8918	-1.5491	-1.968	199	C	-7.5689	-4.7738	2.14467
91	C	10.9406	-1.8957	-2.9987	200	C	-7.1693	-3.5512	2.81746
92	C	8.34887	-2.9882	-3.4599	201	C	-6.2198	-2.7095	2.23552
93	C	9.47464	-3.8713	-3.1919	202	C	-6.4128	-0.7182	-1.8086
94	C	10.7433	-3.3351	-2.9661	203	C	-5.9529	-0.7273	1.00783
95	C	11.5841	-3.8793	-1.9097	204	C	-8.3087	-4.7573	-2.4042
96	C	12.297	-2.773	-1.2911	205	C	-10.816	2.83205	0.33165
97	C	12.5212	-2.7693	0.08416	206	H	-11.119	3.1248	-0.6806
98	C	12.3502	-1.541	0.84609	207	H	-11.682	2.93221	0.98781
99	C	11.7737	-1.8776	2.12745	208	C	-8.6109	3.16431	-0.0221

100	C	10.8234	-1.024	2.69687	209	H	-8.8369	3.42375	-1.0641
101	C	6.89442	0.32731	1.01656	210	N	-9.7193	3.68149	0.76194
102	C	8.1413	-3.5296	3.15999	211	C	-9.5706	3.84355	2.21924
103	C	7.05601	-2.6906	2.90229	212	H	-10.546	3.63044	2.66627
104	C	6.0939	-3.0468	1.86882	213	H	-8.8846	3.10448	2.65352
105	C	6.25871	-4.2242	1.13795	214	C	-9.1622	5.26969	2.58475
106	C	7.3873	-5.0985	1.4125	215	H	-8.2129	5.55293	2.12152
107	C	9.73359	-4.9551	2.17381	216	H	-9.9234	5.97449	2.23775
108	C	10.4463	-3.8496	2.78779	217	H	-9.0591	5.37279	3.6711
109	C	9.46238	-2.9631	3.39208	218	H	8.83681	3.42367	1.06419

Table S6. Atomic coordinates of the optimized dicationic structure of **IT2** using DFT at the B3LYP-D3BJ/6-31G* level. The calculated Gibbs free energy is -8279.257101 hartree at the dicationic state.

No.	Atom	X	Y	Z	No.	Atom	X	Y	Z
1	C	-7.1409	2.28942	-1.0604	110	C	11.6035	-2.4005	3.12222
2	C	-6.6369	2.28104	0.23142	111	C	10.2299	-2.0986	2.7563
3	C	-5.231	2.27599	0.23843	112	C	9.52903	-2.9593	1.90791
4	C	-4.6495	2.26609	-1.0524	113	C	8.82123	-3.2399	-0.312
5	C	-2.7684	2.25999	0.29748	114	C	8.82951	-2.661	-1.5887
6	C	-3.2558	2.24751	-1.0196	115	C	9.75222	-3.1431	-2.6022
7	H	-7.2572	2.28559	1.11572	116	C	10.6411	-4.1734	-2.2934
8	S	-5.8794	2.27486	-2.2795	117	C	12.0175	-4.1079	-2.7456
9	S	-4.0211	2.27748	1.51634	118	C	12.8623	-4.6677	-1.7007
10	C	-2.1162	2.26367	-2.0229	119	C	14.1095	-4.1056	-1.4377
11	C	-1.3475	2.26067	0.3085	120	C	14.5546	-3.9315	-0.0635
12	C	-0.9121	2.26752	-1.063	121	C	14.3137	-2.2841	2.25119
13	C	0.42496	2.25715	-1.3767	122	C	15.1717	-1.8743	1.1489
14	C	-0.4249	2.25706	1.37675	123	C	15.2858	-2.6805	0.016
15	C	0.91216	2.26749	1.06304	124	C	15.2949	-2.0786	-1.3128
16	C	1.34764	2.26071	-0.3085	125	C	14.5685	-2.9591	-2.2077
17	H	0.76193	2.2448	-2.4079	126	C	13.7578	-2.4193	-3.2096
18	H	-0.7618	2.24465	2.40794	127	C	12.4558	-3.0059	-3.4838
19	C	7.14102	2.28953	1.06045	128	C	11.5338	-1.9286	-3.8004
20	C	6.63695	2.2812	-0.2314	129	C	10.2077	-1.996	-3.3732
21	C	5.23105	2.27612	-0.2384	130	C	8.69245	-2.4036	0.86514
22	C	4.64954	2.26614	1.05243	131	C	8.71218	-1.2231	-1.7363
23	C	2.76846	2.26007	-0.2975	132	C	12.428	-4.9106	0.71846
24	C	3.25586	2.24753	1.01963	133	C	10.5893	3.26006	1.88711
25	H	7.25733	2.2858	-1.1157	134	H	10.5852	3.14276	2.97676
26	S	5.87949	2.27488	2.27953	135	H	11.3113	4.03601	1.63042
27	S	4.02119	2.27764	-1.5163	136	C	8.5509	2.3336	1.55127
28	C	2.11627	2.26361	2.02291	137	N	9.25297	3.60542	1.44217
29	C	-2.2548	3.55966	-2.84	138	C	9.15403	4.34805	0.17137
30	C	-1.6745	4.75582	-2.4058	139	H	10.1168	4.84167	0.01078
31	C	-3.0776	3.57681	-3.9741	140	H	9.01263	3.67936	-0.6881
32	C	-1.8963	5.94392	-3.1023	141	C	8.0517	5.40363	0.22266
33	H	-1.0463	4.76515	-1.5211	142	H	7.0687	4.95287	0.38598
34	C	-3.3026	4.76512	-4.6667	143	H	8.24094	6.10436	1.04087
35	H	-3.5264	2.65484	-4.3296	144	H	8.01781	5.96398	-0.7182
36	C	-2.7106	5.95295	-4.2345	145	C	-11.454	1.82507	1.28168
37	H	-1.4321	6.86292	-2.7572	146	C	-10.138	1.22182	1.55356
38	H	-3.9373	4.76041	-5.5477	147	C	-9.3105	0.84332	0.5152
39	H	-2.8822	6.87753	-4.7768	148	C	-9.5423	1.21029	-0.9481
40	C	-2.0528	0.99831	-2.8883	149	C	-10.966	1.86068	-1.2466
41	C	-2.6729	-0.1861	-2.4791	150	C	-11.873	1.99869	-0.0205
42	C	-1.2935	0.99626	-4.0658	151	C	-12.374	1.34195	2.294
43	C	-2.5473	-1.3501	-3.2383	152	C	-11.646	0.45926	3.19085
44	H	-3.2566	-0.2058	-1.5642	153	C	-10.269	0.38822	2.73523
45	C	-1.1645	-0.1675	-4.8211	154	C	-9.5613	-0.8115	2.83363
46	H	-0.8225	1.91341	-4.4052	155	C	-8.6126	-0.4105	0.5992
47	C	-1.7929	-1.3449	-4.4107	156	C	-9.296	-0.133	-1.617
48	H	-3.0412	-2.2601	-2.911	157	C	-10.108	-0.6613	-2.6037

49	H	-0.5793	-0.1518	-5.7357	158	C	-11.425	-0.0653	-2.8853
50	H	-1.6977	-2.2494	-5.0036	159	C	-11.862	1.02517	-2.1586
51	C	2.25484	3.55955	2.84007	160	C	-13.218	1.06581	-1.6958
52	C	1.67452	4.75572	2.40591	161	C	-13.225	1.66769	-0.3711
53	C	3.07769	3.57666	3.97421	162	C	-14.137	1.22267	0.59411
54	C	1.89627	5.94379	3.10253	163	C	-13.7	1.05569	1.96242
55	H	1.0463	4.76509	1.52125	164	C	-12.266	-0.6735	3.7156
56	C	3.30266	4.76494	4.66688	165	C	-13.644	-0.9785	3.35746
57	H	3.5265	2.65468	4.32961	166	C	-14.345	-0.132	2.4999
58	C	2.71062	5.95278	4.23468	167	C	-15.188	-0.6945	1.45507
59	H	1.43206	6.8628	2.7575	168	C	-15.054	0.14302	0.27655
60	H	3.9373	4.76019	5.54782	169	C	-15.047	-0.4351	-0.9995
61	H	2.88214	6.87733	4.77709	170	C	-14.12	0.04184	-2.0086
62	C	2.0529	0.9982	2.88827	171	C	-13.668	-1.0942	-2.7808
63	C	2.67302	-0.1862	2.47898	172	C	-12.337	-1.148	-3.2016
64	C	1.2936	0.99605	4.06575	173	C	-8.6	-1.0127	-0.7249
65	C	2.54745	-1.3503	3.23815	174	C	-11.497	-4.4322	-1.7265
66	H	3.25675	-0.2058	1.56409	175	C	-10.176	-4.1495	-1.38
67	C	1.16464	-0.1677	4.82099	176	C	-9.7352	-4.325	-0.0043
68	H	0.82255	1.91317	4.40517	177	C	-10.632	-4.7762	0.96357
69	C	1.79303	-1.3452	4.41054	178	C	-12.004	-5.081	0.60081
70	H	3.04145	-2.2602	2.91075	179	C	-13.73	-4.3238	-0.993
71	H	0.5794	-0.1521	5.73552	180	C	-13.607	-3.483	-2.1722
72	H	1.69791	-2.2497	5.00331	181	C	-12.224	-3.5442	-2.6227
73	C	11.4539	1.82507	-1.2817	182	C	-11.604	-2.4005	-3.1222
74	C	10.138	1.22186	-1.5536	183	C	-10.23	-2.0987	-2.7563
75	C	9.31054	0.84339	-0.5152	184	C	-9.5291	-2.9593	-1.9079
76	C	9.54232	1.21035	0.94812	185	C	-8.8213	-3.24	0.31198
77	C	10.966	1.86068	1.24658	186	C	-8.8296	-2.6611	1.58873
78	C	11.8728	1.99867	0.0205	187	C	-9.7523	-3.1432	2.60222
79	C	12.3744	1.34191	-2.294	188	C	-10.641	-4.1734	2.29341
80	C	11.6462	0.45925	-3.1908	189	C	-12.018	-4.1079	2.7456
81	C	10.2687	0.38826	-2.7352	190	C	-12.862	-4.6677	1.70064
82	C	9.56129	-0.8114	-2.8336	191	C	-14.11	-4.1055	1.4377
83	C	8.61262	-0.4104	-0.5992	192	C	-14.555	-3.9314	0.06349
84	C	9.29601	-0.133	1.617	193	C	-14.314	-2.284	-2.2512
85	C	10.1078	-0.6612	2.60368	194	C	-15.172	-1.8742	-1.1489
86	C	11.4246	-0.0653	2.88531	195	C	-15.286	-2.6803	-0.016
87	C	11.8624	1.02514	2.15864	196	C	-15.295	-2.0784	1.31277
88	C	13.2179	1.06574	1.69576	197	C	-14.569	-2.959	2.20768
89	C	13.2254	1.66762	0.37108	198	C	-13.758	-2.4192	3.2096
90	C	14.1368	1.22257	-0.5941	199	C	-12.456	-3.0058	3.48383
91	C	13.6999	1.05561	-1.9624	200	C	-11.534	-1.9286	3.80035
92	C	12.2663	-0.6735	-3.7156	201	C	-10.208	-1.9961	3.37322
93	C	13.6441	-0.9785	-3.3575	202	C	-8.6925	-2.4037	-0.8651
94	C	14.3453	-0.1321	-2.4999	203	C	-8.7122	-1.2232	1.73626
95	C	15.1884	-0.6946	-1.4551	204	C	-12.428	-4.9106	-0.7185
96	C	15.0539	0.14289	-0.2766	205	C	-10.589	3.26005	-1.8871
97	C	15.0468	-0.4352	0.99951	206	H	-10.585	3.14275	-2.9767
98	C	14.1205	0.04173	2.00856	207	H	-11.311	4.03602	-1.6304
99	C	13.6675	-1.0943	2.78076	208	C	-8.5508	2.33351	-1.5513
100	C	12.3369	-1.148	3.20161	209	H	-8.5261	2.10084	-2.6234

101	C	8.59992	-1.0126	0.72489	210	N	-9.2528	3.60536	-1.4422
102	C	11.4968	-4.4322	1.72644	211	C	-9.1539	4.34797	-0.1713
103	C	10.1759	-4.1495	1.37998	212	H	-10.117	4.84163	-0.0108
104	C	9.73508	-4.325	0.00425	213	H	-9.0125	3.67927	0.68815
105	C	10.6322	-4.7762	-0.9636	214	C	-8.0515	5.4035	-0.2226
106	C	12.0039	-5.081	-0.6008	215	H	-7.0685	4.95271	-0.3859
107	C	13.73	-4.3239	0.99301	216	H	-8.2407	6.10425	-1.0408
108	C	13.6065	-3.4831	2.17216	217	H	-8.0176	5.96386	0.71827
109	C	12.2239	-3.5443	2.62265	218	H	8.52619	2.10092	2.6234

SI References

1. M. Maggini, G. Scorrano, M. Prato, Addition of azomethine ylides to C_{60}: synthesis, characterization, and functionalization of fullerene pyrrolidines. *J. Am. Chem. Soc.* **115**, 9798-9799 (1993).
2. C. Wang *et al.*, Aggregation promotes charge separation in fullerene-indacenodithiophene dyad. *Nat. Commun.* **15**, 5681 (2024).
3. M. Izquierdo *et al.*, All-fullerene electron donor-acceptor conjugates. *Angew. Chem. Int. Ed.* **58**, 6932-6937 (2019).
4. Y. Chai *et al.*, Interfullerene electronic interactions and excited-state dynamics in fullerene dumbbell conjugates. *J. Am. Chem. Soc.* **145**, 14190-14195 (2023).
5. A. Weller, Photoinduced electron transfer in solution: exciplex and radical ion pair formation free enthalpies and their solvent dependence. *Z. Phys. Chem.* **133**, 93-98 (1982).
6. M. Orazietti, M. Kuss-Petermann, P. Hamm, O. S. Wenger, Light-driven electron accumulation in a molecular pentad. *Angew. Chem. Int. Ed.* **55**, 9407-9410 (2016).
7. Y. Chai *et al.*, In situ switching of photoinduced electron transfer direction by regulating the redox state in fullerene-based dyads. *J. Am. Chem. Soc.* **142**, 4411-4418 (2020).
8. D. M. Guldi, M. Prato, Excited-state properties of C_{60} fullerene derivatives. *Acc. Chem. Res.* **33**, 695-703 (2000).
9. T. Lu, F. Chen, Multiwfn: a multifunctional wavefunction analyzer. *J Comput Chem* **33**, 580-592 (2012).
10. S. Grimme, J. Antony, S. Ehrlich, H. Krieg, A consistent and accurate ab initio parametrization of density functional dispersion correction (DFT-D) for the 94 elements H-Pu. *J. Chem. Phys.* **132**, 154104 (2010).
11. M. J. Frisch*, et al.,* Gaussian 16 (Revision A.03) (Gaussian Inc., Wallingford, CT, 2016).
12. P. E. Hartnett *et al.*, Influence of anion delocalization on electron transfer in a covalent porphyrin donor-perylenediimide dimer acceptor system. *J. Am. Chem. Soc.* **139**, 749-756 (2017).
13. H. Imahori *et al.*, Modulating charge separation and charge recombination dynamics in porphyrin-fullerene linked dyads and triads: Marcus-normal versus inverted region. *J. Am. Chem. Soc.* **123**, 2607-2617 (2001).
14. S. Fukuzumi, K. Ohkubo, T. Suenobu, Long-lived charge separation and applications in artificial photosynthesis. *Acc. Chem. Res.* **47**, 1455-1464 (2014).
15. D. S. Achilleos *et al.*, Solar reforming of biomass with homogeneous carbon dots. *Angew. Chem. Int. Ed.* **59**, 18184-18188 (2020).
16. Q. Zhou, Y. Guo, Y. Zhu, Photocatalytic sacrificial H_2 evolution dominated by micropore-confined exciton transfer in hydrogen-bonded organic frameworks. *Nat. Catal.* **6**, 574-584 (2023).
